# Food, health, and complexity: towards a conceptual understanding to guide collaborative public health action

**DOI:** 10.1186/s12889-016-3142-6

**Published:** 2016-06-08

**Authors:** Shannon E. Majowicz, Samantha B. Meyer, Sharon I. Kirkpatrick, Julianne L. Graham, Arshi Shaikh, Susan J. Elliott, Leia M. Minaker, Steffanie Scott, Brian Laird

**Affiliations:** School of Public Health and Health Systems, University of Waterloo, 200 University Ave. West, Waterloo, N2L 3G1 ON Canada; Social Development Studies, Renison University College-University of Waterloo, 240 Westmount Road North, Waterloo, N2L 3G4 ON Canada; Department of Geography & Environmental Management, University of Waterloo, 200 University Ave. West, Waterloo, N2L 3G1 ON Canada; Propel Centre for Population Health Impact, University of Waterloo, 200 University Ave. West, Waterloo, N2L 3G1 ON Canada

**Keywords:** Public health, Public policy, Health policy, Population-based planning, Foodborne diseases, Food allergy, Food insecurity, Dietary contamination, Obesity

## Abstract

**Background:**

What we eat simultaneously impacts our exposure to pathogens, allergens, and contaminants, our nutritional status and body composition, our risks for and the progression of chronic diseases, and other outcomes. Furthermore, what we eat is influenced by a complex web of drivers, including culture, politics, economics, and our built and natural environments. To date, public health initiatives aimed at improving food-related population health outcomes have primarily been developed within ‘practice silos’, and the potential for complex interactions among such initiatives is not well understood. Therefore, our objective was to develop a conceptual model depicting how infectious foodborne illness, food insecurity, dietary contaminants, obesity, and food allergy can be linked via shared drivers, to illustrate potential complex interactions and support future collaboration across public health practice silos.

**Methods:**

We developed the conceptual model by first conducting a systematic literature search to identify review articles containing schematics that depicted relationships between drivers and the issues of interest. Next, we synthesized drivers into a common model using a modified thematic synthesis approach that combined an inductive thematic analysis and mapping to synthesize findings.

**Results:**

The literature search yielded 83 relevant references containing 101 schematics. The conceptual model contained 49 shared drivers and 227 interconnections. Each of the five issues was connected to all others. Obesity and food insecurity shared the most drivers (*n* = 28). Obesity shared several drivers with food allergy (*n* = 11), infectious foodborne illness (*n* = 7), and dietary contamination (*n* = 6). Food insecurity shared several drivers with infectious foodborne illness (*n* = 9) and dietary contamination (*n* = 9). Infectious foodborne illness shared drivers with dietary contamination (*n* = 8). Fewer drivers were shared between food allergy and: food insecurity (*n* = 4); infectious foodborne illness (*n* = 2); and dietary contamination (*n* = 1).

**Conclusions:**

Our model explicates potential interrelationships between five population health issues for which public health interventions have historically been siloed, suggesting that interventions targeted towards these issues have the potential to interact and produce unexpected consequences. Public health practitioners working in infectious foodborne illness, food insecurity, dietary contaminants, obesity, and food allergy should actively consider how their seemingly targeted public health actions may produce unintended positive or negative population health impacts.

**Electronic supplementary material:**

The online version of this article (doi:10.1186/s12889-016-3142-6) contains supplementary material, which is available to authorized users.

## Background

Food and health are intimately intertwined: what we eat simultaneously impacts our exposure to foodborne pathogens [1–4], allergens [5–9], and environmental contaminants [10, 11], our nutritional status [12, 13], body composition [14–16], mental health [17–19], risks for and the progression of chronic diseases [20–22], and other outcomes. Furthermore, what we eat is influenced by a complex web of drivers, such as socioeconomic status [23–25], food security [26, 27], preferences [28–30], culture [31–34], politics [35–37], economics [38–40], trade [41, 42], industry [43–45], legislation [46, 47], and our built [48–50] and natural [51–53] environments.

To date, public health initiatives aimed at improving food-related population health outcomes have primarily been developed within ‘practice silos’ (e.g., chronic disease prevention efforts typically developed independently from food safety activities), and their potential to interact or have unintended consequences has been largely ignored. As is the case in any system, ‘solutions’ to one issue may create new problems for another [54]. With respect to food and health, for example, food safety measures to limit microbial growth, such as the addition of salt [55, 56], can pose chronic disease risks [57, 58], and the development of urban gardens to improve food security [59–61] can increase exposure to a variety of contaminants [62–65]. Therefore, understanding the potential for complex interactions among public health initiatives aimed at improving specific aspects of population health (here, as related to food) is imperative to understanding how such efforts may influence one another, or influence seemingly unrelated population health outcomes, in unexpected ways.

Systems thinking offers public health practitioners a paradigm for framing issues that explicates complexity and interrelationships among parts of a whole, and facilitates identification of less obvious influences and consequences [66, 67]. Systems-informed public health initiatives exist in the realm of food and health; perhaps the most well-known is the Foresight Obesity System Map [68], an in-depth exploration of the complex web of social, economic, biological, psychological, and other drivers of a single food-related population health issue, obesity. In addition to exploring the myriad of drivers for one issue, the bilateral connectivity between issues has been explored, for example between obesity and food security [69]. Such bilateral explorations of food-health issues have also been expanded to involve system actors (i.e., those whose actions influence the parts within the system) from non-health sectors. For example, one local public health agency integrated the economic viability of agriculture with community food security, diet, and nutrition considerations to generate a municipal-level healthy community food system approach [70]. However, there are no conceptual models that support thinking systemically about multiple food-related population health issues in concert, to provide a roadmap for considering how targeted public health initiatives may interact in unexpected ways or have unintended consequences. Therefore, the objective of this study was to develop a conceptual model depicting how multiple food-related population health issues can be linked via shared drivers, illustrating the potential for complex interactions, with the ultimate goal of supporting collaboration across public health practice silos to guide informed interventions.

## Methods

We applied a complex adaptive systems (CAS) lens to the concept of population health as related to food and diet. A CAS lens suggests that a given population health outcome or issue is an emergent property of an underlying system of inter-related drivers including political, environmental, social, biological, and other factors [71]. We developed a conceptual model depicting the shared drivers of five population health issues related to food (hereafter called ‘issues’): infectious foodborne illness, food insecurity, dietary contaminants, obesity, and food allergy. We selected these because (a) they are important public health issues for which there is significant investment in prevention and improvement, and (b) we hypothesized that they had shared drivers, and we assembled our research team to provide expert knowledge in these areas. We defined ‘drivers’ broadly, as factors with the potential to impact one or more issues, and included drivers across scales (from individual to societal) and types of associations (e.g., direct causes, higher-level proxies for more complex pathways). The conceptual model was developed by identifying drivers from the peer-reviewed literature and synthesizing shared drivers into a common model, using a modified thematic synthesis approach [72] that combined a systematic search process, inductive thematic analysis [73], and mapping to synthesize findings.

In March 2015, we searched the peer-reviewed literature in MEDLINE (via PubMed) for English-language reviews published in the last five years, using the search terms shown (Table 1). We selected only review articles for inclusion because initial topic-specific searches returned between 800 and 54,000 articles per topic. We designed the search to identify recent, evidence-based descriptions of a range of drivers of the five chosen issues; the search was not intended to produce a comprehensive nor exhaustive list of drivers, but rather to identify a range of drivers, including possible shared drivers. Additional articles were identified via citation cross-referencing and from authors’ areas of expertise. Because we included all types of review articles, including traditional narrative reviews, and because there is lack of consensus on how to assess quality among such heterogeneous types of reviews, we did not assess article quality.

**Table 1:** Search strings used to identify English-language reviews describing drivers of five food-related population health issues

Food-related population health issue	Search conducted
Obesity	(system OR complexity OR model OR driver OR influence OR determinant OR “risk factor”) AND (weight OR obes* OR (food AND (neighborhood OR neighbourhood)) OR “food environment” OR “nutrition environment” OR “food retail” OR “food desert” OR “food store” OR “food access”)
Food allergy	(system OR complexity OR model OR driver OR influence OR determinant OR “risk factor”) AND ((food AND allerg*) OR (food AND anaphylaxis))
Infectious foodborne illness	(system OR complexity OR model OR driver OR influence OR determinant OR “risk factor”) AND (“food safety” OR “foodborne disease” OR “food-borne disease” OR “foodborne illness” OR “food-borne illness” OR “food poisoning” OR (food AND pathogen) OR (food AND infection))
Food insecurity	(system OR complexity OR model OR driver OR influence OR determinant OR “risk factor”) AND (“food security” OR “food insecurity” OR “food system” OR hunger OR “food deprivation” OR “food affordability” OR “food unaffordability” OR “food accessibility” OR “food inaccessibility” OR “food sufficiency” OR “food insufficiency” OR “food access” OR “food poverty”)
Dietary contaminants^a^	(system OR complexity OR model OR driver OR influence OR determinant OR “risk factor”) AND ((food AND toxin) OR (diet AND toxin) OR (food AND toxicant) OR (diet AND toxicant) OR (food AND pollutant) OR (diet AND pollutant) OR (food AND contaminant) OR (diet AND contaminant) OR (food AND metal*) OR (diet AND metal*) OR (food AND chemical*) OR (diet AND chemical*) OR (food AND (PAH OR “polycyclic aromatic hydrocarbon”)) OR (diet AND (PAH OR “polycyclic aromatic hydrocarbon”)) OR (food AND (POP OR “persistent organic pollutant”)) OR (diet AND (POP OR “persistent organic pollutant”)) OR (food AND (EDC OR “endocrine disrupting chemical”)) OR (diet AND (EDC OR “endocrine disrupting chemical”)) OR (food AND mercury) OR (diet AND mercury) OR (food AND cadmium) OR (diet AND cadmium))

^*^This symbol indicates that the truncation search feature was used in order to capture all variations of this search term

^a^Search terms were included to yield a cross-section of key dietary contaminants within environmental public health

Search results were combined in a RefWorks database (2015, ProQuest LLC) and duplicates eliminated.

**Fig. 1:**
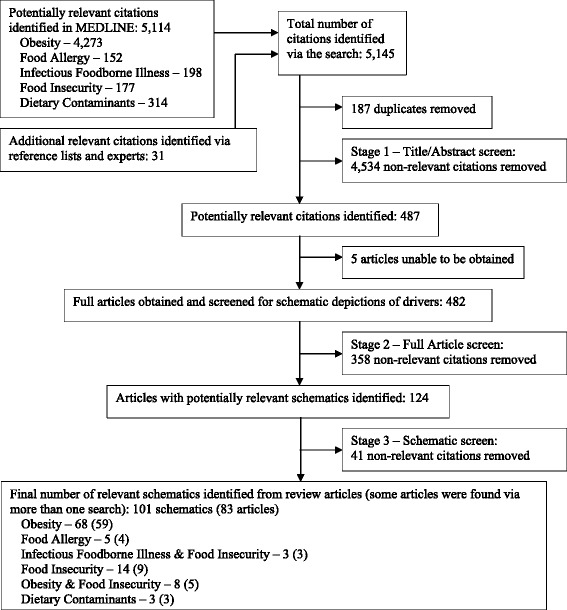
Search results for English-language reviews (January 2010-March 2015) of five food-related population health issues

Two reviewers conducted the first relevance screen (Fig. 1, Stage 1). Both independently reviewed the first 87 articles; given high reviewer agreement (kappa = 0.891 [95 % C.I. 0.786, 0.995]), the remaining articles were screened by one reviewer per reference, using the title, and abstract if available. Articles were considered relevant and were included if they: explicitly identified drivers of one or more of the five population health issues, or depicted the links between two or more of the issues; pertained to human populations (including specific sub-populations); and were relevant to developed country contexts. Developing country contexts were considered out-of-scope for this study because social, cultural and political drivers differ greatly between developing and developed contexts. Articles pertaining solely to the following were excluded: animal or plant populations; animal models; development of laboratory or measurement techniques; methods for screening or surveillance for the population health issue; diagnosis, clinical characteristics, management, treatment, or impacts (including costs) of the issue; or details of chemical, hormonal, microbiological, cellular, or molecular properties or processes (including pathogenesis).

Applying a CAS lens, full text articles identified via the first screening were obtained, and screened, by a single reviewer per article (Fig. 1, Stage 2), to identify those containing any schematic representation that depicted drivers of one or more of the issues. The remaining articles were then screened, by a single reviewer per article (Fig. 1, Stage 3), to identify those containing causal diagrams, conceptual models, or similar schematics that included some visual representation of the relationships between the driver(s) and issue(s). Articles without such representations were excluded. Types of visual representations included were: causal loop diagrams; conceptual representations that showed or described either the directionality of relationships, or strength of effect of drivers on outcomes; and socio-ecological frameworks that depicted specific driver(s) and their relative relationships to the issue(s). Schematics were included if they depicted either the broad issue (e.g., obesity), or a specific case of the broad issue (e.g., adipose tissue proliferation). Schematics that named drivers without depicting or describing their relationships to the issue (e.g., a bullet-point list of drivers with no relationships shown or described) were excluded.

To synthesize the final set of schematics into a conceptual model, we extracted those drivers that were common to two or more issues (as well as their associations with the issues) and combined the driver-issue associations into a single model via an inductive thematic analysis, as follows. First, we familiarized ourselves with all schematics, specifically the types of drivers and ways that a given driver (e.g., global warming) might be differentially expressed (e.g., “warming climate”, “permafrost thaw”). From our identified schematics, we created an initial working list of drivers within each of the five issues. Drivers found only within one issue’s literature (e.g., physical activity factors found only in the obesity literature) were omitted from further analysis.

We then created a codebook containing preliminary names and descriptions for the drivers. Specific wording in a given schematic (e.g., “high fat diet and gut microbiota”) relevant to more than one of our drivers (e.g., ‘gut microbiota’ and ‘Western-style diet’) was captured under each relevant driver (see Additional file 1). Two authors then independently reviewed 10 references’ schematics (two from each issue), refined the driver names and descriptions in the codebook accordingly, and compared, discussed, and merged these revisions. The resulting revised codebook was used to code drivers within all schematics, and was iteratively refined during coding, such that the final codebook of drivers (Additional file 1) fully captured the drivers in the identified literature. The codebook (during development and in its final state) was reviewed by research team members from the five issue areas, to ensure that information from the literature was being accurately captured under all relevant drivers, and that names and descriptions developed for each driver adequately reflected the details from the identified literature. Using the final codebook, we identified common drivers from all schematics. Each identified driver was extracted, together with its depicted association with the issue(s) of interest and with any other drivers, and these depictions were merged to produce the conceptual model, in Vensim® PLE Plus for Macintosh (version 6.3; Ventana Systems, Inc.).

Because one issue could be a driver for another issue (e.g., adenovirus infection, under the umbrella of infectious foodborne illness, linked to obesity [74]), these associations were also extracted and included in the conceptual model. Relationships between drivers that were described textually in the schematic instead of visually (e.g., “socio-cultural norms influencing food choice”) were also included in the conceptual model. Because the goal was to depict potential connections between issues, rather than definitively represent causes, we did not assess the strength of association or the type of correlation (i.e., positive [e.g., an ‘increase’ in the driver ‘increases’ the issue], or negative [e.g., an ‘increase’ in the driver ‘decreases’ the issue]) between drivers and issues. Rather, we captured solely whether there was suggestion of a relationship among the five population health issues and their common drivers, that is, that a given driver could lead to, of have influence on, the population health issue(s).

## Results

**Fig. 2:**
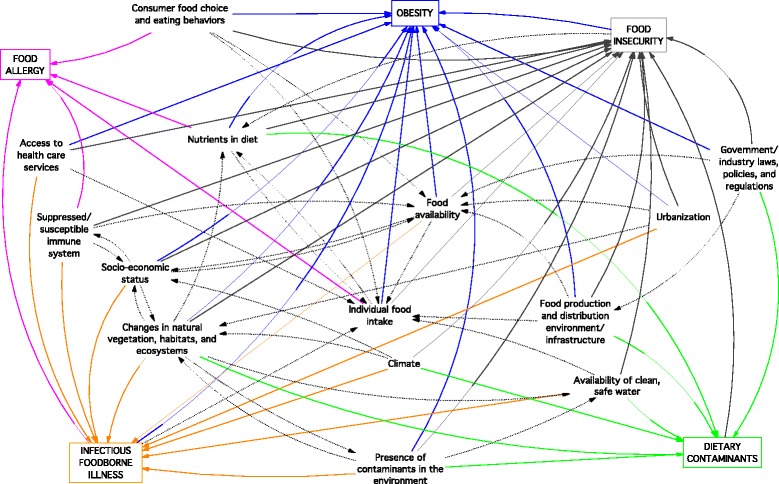
The 14 drivers shared between three or more of the five food-related population health issues

An initial 5145 references were identified from the literature search (Fig. 1). Via screening, we identified 83 relevant references containing 101 schematics; from these, 49 drivers common to two or more of the issues were identified. All drivers, and their specific wording extracted from the literature, are given by issue and reference (Additional file 1). Table 2 shows the 35 drivers shared between only two of the five population health issues [75–149]. Figure 2 depicts the 11 drivers common to three issues (climate [75, 124, 138, 145, 146, 149]; consumer food choice and eating behaviours [76, 86, 91, 97, 109, 112, 115, 117–119, 123–126, 129, 130, 139, 141, 142, 150, 151]; individual food intake [76, 87, 97, 101, 114, 116, 120, 122, 124, 129–131, 139, 141, 143, 151, 152]; food availability [76, 100, 101, 104, 112, 113, 117, 123–125, 128–130, 136, 138, 146]; suppressed/susceptible immune system [97, 124, 136, 146]; socioeconomic status [75, 76, 93, 98, 100, 117, 123, 124, 127, 128, 130, 137, 138, 146]; availability of clean, safe water [75, 124, 130, 148]; urbanization [113, 127, 129, 138, 145]; access to health care services [75, 100, 109, 124, 130]; food production and distribution environment and infrastructure [86, 129–131, 153]; and government and industry laws, policies, and regulations [100, 101, 109, 126, 127, 130, 132, 133, 135, 138, 153]), and the three drivers common to four issues (nutrients in diet [83, 93, 97, 129, 134, 148, 154–156]; changes in vegetation, habitats, and ecosystems [100, 123, 124, 133, 138, 145–148]; presence of contaminants in the environment [75, 79, 80, 89, 123, 124, 137, 138, 147, 148, 153]). No drivers were common to all five issues.

**Table 2:** The 35 drivers shared between only two of the five food-related population health issues

	Infectious foodborne illness	Dietary contamination	Food allergy	Food insecurity
Obesity	Population demographics [75, 76]	Diet [77–80]	Gut microbiota [77–79, 81–97]	Food prices and affordability [98, 117, 123–128]
			Genetics [78, 79, 82, 84, 86, 90, 94, 97–104]	Food environments [76, 100, 102, 104, 119, 126–128]
			Epigenetics [81, 86, 89, 97, 105–109]	Social norms [113, 116, 126, 129–133]
			Western-style diet [83, 84, 89–92, 94, 98, 110–116]	Types of foods available within schools and daycares [76, 100, 125–127, 133–135]
			Age [76, 84, 93, 97, 100, 108, 109, 117, 118]	Health status [79, 117, 124, 125, 131, 135–137]
			Caesarean birth [79, 81, 90, 94, 95]	Sex and gender [76, 79, 84, 100, 109, 117, 127, 128]
			Use of antibiotics [79, 84, 90, 94, 95]	Ethnicity [76, 109, 118, 128]
			Early life feeding [76, 79, 81, 86, 93–95, 97, 98, 100, 102, 107, 108, 116, 117, 119–121]	Culture [101, 117, 128–130, 132, 133, 138, 139]
			Maternal-fetal interaction [81, 89, 95, 98, 104–106, 122]	Globalization and increasing global trade [113, 129, 130]
				The economic environment [113, 123, 124, 129, 132, 133]
				Food marketing and advertising [86, 98, 101, 112, 124–128, 130]
				Inter-personal influences and supports [76, 100, 101, 109, 117, 125, 126, 130, 131, 133, 135, 138, 139]
				Food skills and knowledge [76, 86, 93, 101, 116, 117, 123, 125–127, 130, 138]
				Household/family structure and dynamics [76, 98, 100, 101, 116, 117, 119, 121, 125, 126, 129, 130, 133, 135, 137–140]
				Built environment [86, 98, 113, 123, 127, 132, 133, 135, 138, 139]
				Community dynamics and well-being [76, 100, 123, 133, 137]
				Time and resources needed to eat ‘healthy’ [101, 117, 123, 125, 141–144]
Infectious foodborne illness	-	Global warming [75, 145–147]	Changes in exposure to infectious diseases [75, 94, 96]	(none)
		Precipitation [75, 145–148]		
		Spatial co-existence of people with fauna [145, 147, 149]		
		Agricultural intensification [145, 147]		
Dietary contaminants	-	-	(none)	Traditional foods and diet [123, 138, 147]
				The food supply [124, 128, 130, 133, 147]
Food allergy	-	-	-	(none)

The full diagram showing the 49 drivers, their links with the five issues, and their 227 interconnections is given in Additional file 2. Tree diagrams presenting the same links, but by individual issue, are also given (Additional file 3). Of the 49 drivers, 14 were directly associated with dietary contaminants, 14 with food allergy, 15 with infectious foodborne illness, 33 with food security, and 39 with obesity. Obesity and food insecurity shared the most drivers (*n* = 28). Obesity shared several drivers with food allergy (*n* = 11), infectious foodborne illness (*n* = 7), and dietary contamination (*n* = 6). Food insecurity shared several drivers with infectious foodborne illness (*n* = 9) and dietary contamination (*n* = 9). Infectious foodborne illness shared drivers with dietary contamination (*n* = 8). Fewer drivers were shared between food allergy and: food insecurity (*n* = 4); infectious foodborne illness (*n* = 2); and dietary contamination (*n* = 1).

## Discussion

We merged visual schematics extracted from peer-reviewed literature reviews to produce a conceptual model showing how infectious foodborne illness, food insecurity, dietary contaminants, obesity, and food allergy are connected via 49 shared drivers. Although none of the identified drivers were surprising or unexpected in and of themselves, synthesizing the drivers into a common model provided new insights into potential interrelationships between issues for which interventions have historically been siloed in public health practice. Because of these connections, interventions targeted towards individual issues that impact the identified drivers have the potential to ‘ripple’ through the system, interacting and producing unexpected consequences. It therefore behooves individuals working in these areas to actively consider how their seemingly targeted activities (e.g., enforcing municipal sanitation requirements for food premises) may have unintended impacts in the larger system (e.g., emergency food provision suffering due to the need to expend resources on training rather than service delivery). Our conceptual model offers a heuristic for such systemic thinking that can aide future collaboration across public health practice areas to guide informed interventions. Of course, we recognize that complex conceptual models such as ours can produce “despair and retreat” [131], leaving practitioners unclear about how to operationalize findings within their day-to-day activities. To this end, we offer several concrete applications of this model, as follows.

First, this model can be used to more fully understand the range of drivers impacting a single population health issue, particularly within specific contexts. For example, practitioners wishing to identify drivers and points of intervention to improve community food security can use this conceptual model as an evidence-based draft causal loop diagram to start a group model building process [157–159], thereby layering tacit knowledge from practitioners, industry, community groups, and other stakeholders and system actors onto literature-based evidence. In doing so, it will be important to ask: ‘what other key drivers and relationships do we need to add to the model, given our specific context?’; ‘what drivers and relationships are irrelevant to our context?’; and, ‘what drivers and relationships are too vague and need to be more detailed?’. Creating a more complete model, however, must be balanced against utility, since increasingly complex conceptualizations become more difficult to understand and apply. Nevertheless, this model is a useful starting point for those wishing to identify driving forces for a given issue, to “[transcend] silos to find solutions” [160].

Second, this model can be used to explore how a single driver can widely impact multiple issues, ultimately revealing high leverage drivers deserving concerted public health effort. For example, in our model, socio-economic status was directly (*n* = 3) or indirectly (*n* = 2) related to all five population health issues. Evidence indicates that low-income families of food allergic children may have difficulty managing the child’s allergy because of a lack of affordable medication and the perceived high cost of allergen-free foods [161–163], and that low-income individuals are also at risk for obesity [100, 117, 127, 137], contracting foodborne disease [146, 164], and food insecurity [123, 124, 128, 130, 133]. An association between socio-economic status and exposure to dietary contaminants may exist, but is less clear [165]. Therefore, our model highlights the importance of considering the impacts of socio-economic status in addressing any of the food-health issues discussed here, concurrent with prior calls to focus on the underlying socioeconomic determinants of health [166]. Other potential high leverage drivers are those that directly influence multiple issues; here, we identified three (nutrients in diet; changes in vegetation, habitats, and ecosystems; presence of contaminants in the environment) that were directly associated with four of the five population health issues. A more detailed exploration of the specific ways in which these drivers can influence population health (e.g., ‘which nutrients in the diet may help mitigate or reduce food insecurity, allergy, obesity, and dietary contamination?’) may help identify high leverage areas where actions or interventions may yield multiple benefits.

Third, this model can be used to identify relevant systems actors with whom to engage when planning, undertaking, or evaluating public health actions, programs, or policies. Although collaboration is a necessary response to addressing complex problems [167], Trochim et al. [67] identified “supporting dynamic and diverse networks” as a key challenge to effective systems thinking in public health. Our model can be used to identify which diverse, potentially non-traditional individuals to approach, particularly from other areas within public health. For example, individuals developing school food allergy policies can use the model to ask whether actions of public health inspectors, food security advocates, nutritionists, health promotion specialists, environmental or toxicological risk assessors, or other public health actors could impact the planned policies, and therefore whether said individuals should be engaged. At the very least, public health practitioners should invite those with responsibility for other food-health issues to discuss potential impacts of their respective actions. Although public health organizations recognize the importance of engaging other non-health sectors [168], working intra-organizationally across seemingly disparate units (e.g., food safety and obesity) seems less common. Since individuals and dialogue matter within complex systems [131, 169], organizational support for dialogue across public health domain areas is needed, particularly among front line practitioners who understand the contexts and nuances of the food-health issues.

Fourth, this model can be used to explore potential unintended consequences of actions, programs, or policies. Public health practitioners can apply this model to their context-specific practice situations, to assess how their activities might impact the drivers in the model, and thus whether they might inadvertently impact any of the other four issues. For example, food security advocates developing local programs that encourage community gardens and urban agriculture should consider whether these activities have the potential to impact food allergy, infectious foodborne disease, obesity, or dietary contaminants for the population involved. Although there is evidence that community gardens can positively impact food security [61], they can also lead to soil contaminant exposure via increased incidental soil ingestion and accumulation of contaminants within edible plant tissues [62–64, 170]. Whether the detrimental impact on contaminant exposure exceeds the beneficial impact on food security will inevitably be context-specific, again underscoring the need for front line practitioners to engage across the issues, to co-develop public health actions that minimize risk while striving to improve population health. In addition to identifying inadvertent impacts of an activity on population health, this model can help identify how diverse public health actions, programs, or policies may act in synergy or antagonistically. For example, food security advocates and food allergy policy makers interested in foods in schools could work together to ensure that programs that improve access to nutritious foods (e.g., school fruit and vegetable programs) and restrictions on allergic foods in classrooms are co-developed, ensuring that food allergic and food insecure children are not negatively impacted; additionally, engaging food safety experts in such plans will help minimize infectious foodborne illness risks that may inadvertently result from shifting the types of foods available and allowable in school environments.

Finally, this model may help reframe how future public health actions are evaluated and issues prioritized. Trochim et al. [67] identified priority setting by “analyzing system-wide issues rather than simply ranking disease burden or attributable risk” as a challenge to effective systems thinking in public health. Reframing successful public health initiatives from ones which reduce disease burden for one issue, to ones which do so without negative consequences for other issues, and ultimately to a suite of initiatives that act together to optimize population health across all issues may be useful. This idealistic goal may mean explicitly accepting less-than-optimal health states for individual issues in order to optimize population health overall. However, it is worth acknowledging that – akin to “health in all policies” [171], which gives non-health sectors the responsibility to consider impacts of their activities on health – public health practitioners bear a responsibility to consider “all health in policies”, that is, to explicitly consider other potential health impacts of their planned or current activities. At the very least, activities that target reducing the burden of a single issue without considering potential impacts on the other issues should be challenged to explain their impact on population health as a whole, whether any unintended negative consequences might arise, and what consultation has occurred to mitigate such consequences.

In addition to the model and its potential applications, we also offer a methodological approach for use by public health researchers faced with synthesizing evidence from different domains. We used a modified thematic synthesis [72] to merge and map evidence across five bodies of literature that varied substantially. We found the evidence varied significantly in scale, scope, and the terminology used to describe the drivers, both between and within the five issues’ bodies of literature. Our inductive thematic mapping allowed evidence about drivers to be synthesized across disciplines, despite differences in terminologies and conceptualizations. We also observed that literature on issues underpinned by disciplines with a historically strong biomedical paradigm, or for which specific single causes are known (here, infectious foodborne illness and dietary contamination) had substantially fewer reviews that collated evidence on known social, economic, political, environmental, and other drivers. In contrast, issues such as obesity and food insecurity, for which discrete causal agents do not exist, had strikingly more review articles covering the range of drivers and their relationships to the issue, and therefore yielded the majority of common drivers in our model. Thus, our model may be differentially biased towards these drivers, and future efforts to illuminate the broad range of drivers of infectious foodborne illness and dietary contamination are warranted. Nevertheless, our methodology provides a practical and systematic means of synthesizing evidence present in the peer-reviewed literature that may otherwise remain in silos.

Our results are subject to several limitations that highlight the challenges in synthesizing the vast and varied evidence encountered when investigating significant public health issues with a systems lens. To manage the volume of literature and support creation of a visual conceptual model, we chose to search for schematic representations from published review articles indexed in the database MEDLINE, meaning that we did not include grey literature, and were constrained to articles predominantly from the natural and health sciences. Thus, our conceptual model likely underrepresents important drivers investigated predominantly within the social sciences, such as factors related to food industry marketing. Even within the scope of the natural and health sciences, we recognize that our strategy captured only a subset of the known drivers of our five issues of interest. For example, relationships between food insecurity and diet [172] were not included in our final set of articles, and although our search identified ‘means of food storage and preservation’ as linked to food insecurity [130], it did not identify known links between this driver and infectious foodborne illness [173]. Thus, our conceptual model contains only a portion of the actual drivers of, and inter-relationships among, the five population health issues included here. Another limitation associated with synthesizing a large amount of literature from different disciplines is the variation in terminology, language, scope, and scale, as described above. Our multidisciplinary team was specifically assembled to overcome variations in terminology and language. However, variation in scope and scale across issues’ bodies of literature necessitated limiting our search to key important concepts (e.g., including only some key dietary contaminants), as well as simplification of complex concepts into higher-level drivers, and precluded any assessment of the types of correlations (i.e., positive, negative) and strengths of identified associations. Thus, the drivers presented in our model each comprise what is, in reality, a multifaceted set of variables and relationships of differing influences and strengths. In future, those applying this conceptual model should consider whether including more detail could help (or hinder) population health efforts.

## Conclusions

This model is an evidence-based starting point for researchers and public health practitioners to collaborate across practice areas, specifically infectious foodborne illness, food insecurity, dietary contaminants, obesity, and food allergy. This model has value as a heuristic in approaching these important public health issues systemically, and can help individuals answer questions like: “what unexpected forces may impact my issue?”, “which potentially non-traditional individuals should I be involving in discussions?”, “how might my planned activities have negative impacts on population health?”, and “who should I engage with to minimize unintended consequences?”. In doing so, it is important to recognize that this model is strongly rooted in evidence from the natural and health sciences, that important evidence from the social, economic, and political science realms is likely missing, and that including experts from these missing domains will be important when answering the above questions. This model also suggests that involving other non-health sectors in multi-disciplinary collaborations may be insufficient when addressing complex population health issues, and that collaborations must also include individuals from other seemingly unrelated public health practice areas in order to best optimize policy and program outcomes. In future, research examining the utility of conceptual models such as this one in specific public health practice situations is warranted, particularly as practitioners seek to incorporate systems perspectives into day-to-day activities.

## Abbreviation

CAS, complex adaptive systems

## Additional files

Additional file 1: Drivers of the five population health issues related to food, showing verbatim wording for all instances of the driver extracted from the included literature, with references; extracted wording that fit with more than one driver is underlined. (PDF 260 kb) Additional file 2: The 49 drivers, common to two or more of the five population health issues related to food, as identified from the literature; dashed arrows are interconnections between drivers, and coloured arrows are direct connections to a given population health issue. (PDF 86 kb) Additional file 3: Tree diagrams showing the relevant drivers for each of the five population health issues related to food; drivers in brackets are those that are also found elsewhere in the particular tree. (PDF 207 kb)
