# Supplementary material for: Food, health, and complexity: towards a conceptual understanding to guide collaborative public health action
Source: BMC Public Health. 2016 Jun 8;16:487. doi: 10.1186/s12889-016-3142-6 (PMC4898364; doi:10.1186/s12889-016-3142-6)
Supplement: Additional file 1: — Drivers of the five population health issues related to food, showing verbatim wording for all instances of the driver extracted from the included literature, with references; extracted wording that fit with more than one driver is underlined. (PDF 260 kb) [file 12889_2016_3142_MOESM1_ESM.pdf]

Supplemental Online Appendix A. Drivers of the Five Population Health Issues Related to Food, Showing Verbatim Wording for all Instances of the Driver Extracted from the Included Literature, with References; Extracted Wording that Fit with More than one Driver is Underlined.

**Driver**	**Driver Description**	**Specific Instances of Driver as Worded in the Literature (Reference)**	**References, by Population Health Issue Related to Food**				
			**Obesity**	**Infectious Foodborne Illness**	**Dietary Contaminants**	**Food Allergy**	**Food Insecurity**
Climate	Weather patterns including general climate change	seasonality;[1] climate change, extreme weather;[2] climate effects, climate change;[3] facilitative factors (e.g., water, warmth);[4] climate;[5] climate change[6]		1-3	4		5, 6
Global warming	Increasing temperature in the broader environment (e.g., climate warming) and expressions thereof (e.g., permafrost thawing, snow cover melt)	climate warming;[1] heatwaves/temperature;[2] temperature;[3] permafrost thaw, seasonal snow cover and melt dynamics, sea-ice cover[7]		1-3	7		
Precipitation	Factors related to the pattern and amount of rain, snow, etc.	precipitation;[1, 2] humidity, rainfall;[3] circulation and precipitation patterns;[7] rainfalls[8]		1-3	7, 8		
Gut microbiota	The microbes living in the human/individual's gut, from birth and across the life course, including the microbes that come from diet	healthy gut microbiota, obese microbiota transfer, gut microbiota disbalance;[9] gut microbiota;[10] intestinal microbiota;[11] microbial dysbiosis in the intestinal tract;[12] pre/pro-biotics in diet, gut microbiota changes;[13] altered microbiota;[14] microbiome;[15] influence of the microbiome;[16] microbial community composition & metabolites;[17] composition of gut microbiota;[18] microbiota maturation, microbiota dysbiosis;[19] <u>high fat diet and gut microbiota;</u>[20] microbe-induced obesity, disrupted microbial community;[21] change in microbiota pattern;[22] probiotics/prebiotic food complements;[23] intestinal microflora;[24] alteration of commensal microbiota "dysbiosis";[25] infant gut microbiota, probiotics supplementation;[26] abnormal microbial flora;[27] colonization of commensal microbiota, indigenous microbiota[28]	9-24			25-28	

Genetics	An individual's genetic makeup, including their genetic susceptibilities	genetics;[10, 16, 30] host's genetic background;[13] genetic predisposition;[15, 19, 32] gene-induced obesity;[21] genetically susceptible individual;[25] host genes, genetic programming/impact;[28] genetic factors;[29] genes;[31, 33, 34] role of genetics[35]	10, 13, 15, 16, 19, 21, 29-35			25, 28	
Epigenetics	The influence of the external environment on gene expression	epigenetic changes;[9, 37] epigenetics;[16, 20] epigenetic re-programming;[28, 38] epigenetic mechanisms;[36] epigenetic modifications;[39] gene-environment interactions[40]	9, 16, 20, 36-40			28	
Western-style diet	A typically less healthy diet, characterized as being higher in fat (especially saturated fats), calories, sugar, and refined energy-dense foods	high fat and high fructose dietary pattern;[12] diet consisting of nondigestible carbohydrates and fat;[13] high fat diet and gut microbiota;[20] high fat/high calorie diet;[21] high fat, low fibre diet;[22, 23] change in dietary patterns;[22] Western (high fat) diet;[25] unhealthy food and beverages;[29] excessive intake of western diet;[41, 42] palatability of diet;[43] increased consumption of energy-dense foods, animal products, refined grains and added sugar i.e. sugar-sweetened beverages;[44] consumption of high-fat foods, high-refined carbohydrate diet;[45] palatability/hedonic pleasure drives over-consumption;[46] obesogenic foods and drinks[47]	12, 13, 20-23, 29, 41-47			25	
Diet	Food and drink regularly consumed or provided; diet can be considered in terms of its quality, composition, and effects on health	diet[11, 15, 19, 48]	11, 15, 19		48		
Traditional foods and diet	A traditional Aboriginal diet, i.e. foods originating from local plant or animal resources through hunting, fishing, gathering, or harvesting	loss of traditional knowledge, less traditional foods available;[6] diet transition (less traditional food items, more food originating elsewhere);[7] importance of hunting/fishing/gathering and traditional food practices, barriers to traditional food acquisition[49]			7		6, 49
Nutrients in diet	The nutrient composition of the diet, including having adequate amounts of vitamins and minerals	animals and game, forages uptake, root and leafy vegetables, pulses, cereal uptake, wild fish and seafood, human food intake;[8] decreased nutrient intake and/or nutritional deficiencies (Vit A, D, Zn, Ca, Mg);[12] intakes of extensively reared food producing macronutrient intake;[24] vitamin D deficiency;[27] dietary immunomodulatory variables, dietary factors (lipids, omega 6 & 3, vit A);[28] dietary calcium, vitamin D;[50] better nutrition;[51] whole grain	12, 24, 50-53		8	28	54

		intake, dietary fibre;[52] overnutrition, excessive antioxidants;[53] variations in consumption of nutrition[54]					
Consumer food choice and eating behaviours	The types of food preferred and chosen by consumers, including consumer demand for food and individual decision-making	individual decision-making, demand for food, food quality;[5] consumer behaviour;[16] change in dietary patterns;[22] lifestyle (nutrition);[28, 65] dietary behaviors;[40] palatability of diet;[43] palatability/hedonic pleasure drives over-consumption;[46] dietary change, rationing, and food purchasing patterns;[49] food acceptability;[54] child's eating behavior;[55] changes in eating behavior (food types/preferences);[56] eating habits;[57] eating patterns;[58, 64] food choice;[59] parent food preferences;[60] child's food preferences;[61] desire for energy dense foods;[62] parental dietary behavior;[63] consumer acquisition and demand for food[66]	16, 22, 40, 43, 46, 55-65			28	5, 49, 54, 66
Individual food intake	The quantity of food consumed and absorbed by an individual	nutrient absorption;[5] dietary intake;[17, 32] lifestyle (nutrition);[28, 65] increased feeding frequency/amount, increased energy intake;[45; 56; 69] child's food intake;[47] food intake;[50, 59] overnutrition, excessive antioxidants;[53] variations in consumption of food;[54] parent dietary intake, child's dietary intake;[60] consumption, digestion, metabolism of food, transport of nutrients, utilization of nutrients;[66] increased food intake;[67; 70; 72] changes in eating behavior (food amounts), increased beverage intake;[68] food consumption[71]	17, 32, 45, 47, 50, 53, 56, 59, 60, 65, 67-72			28	5, 54, 66
Food prices and affordability	The cost of food, set within larger context of other resource factors like social supports, cost of living, etc.; includes differential pricing of types of foods (e.g., healthy versus junk food)	Prices;[5] food prices;[29] affordability of food;[49, 61, 74] economic-nutrition resources;[55] consumer nutrition environment (price, promotion, placement, nutrition information);[64] food environment (price of fast and junk food)[73]	29, 55, 61, 64, 73				5, 49, 74
Food environments	A collection of physical, biological and social factors that affect eating habits and patterns; includes food store home, macro, public facility, restaurant, school and daycare, worksite, and other food environments.	availability of healthy foods in stores;[31] obesogenic food environment;[33, 35]accessibility of convenience foods and restaurants;[6] child's home food environment;[63] community nutrition environment (food access and food availability), organizational nutrition environment, consumer nutrition environment (price, promotion, placement, nutrition information), perceived nutrition environment;[64] food	31, 33, 35, 60, 63, 64, 73				74

		environment (availability of fast and junk food vs healthy food);[73] community food system (stores, restaurants, schools, workplaces, etc)[74]					
Food availability	The general availability and accessibility of food, including differential distribution, quantity and types of foods	nutrition food availability;[3] food availability; [5, 55, 66] less sharing, less traditional foods available;[6] availability of healthy foods in stores;[31] food availability & accessibility;[32, 75] abundant food availability;[35] ready availability;[43] changes to food availability;[44] food sharing;[49]differential food availability and accessibility;[54] types of food available in the home;[60] accessibility of food;[61] intra-household distribution of food;[66] community food system (stores, restaurants, schools, workplaces, etc)[74]	31, 32, 35, 43, 44, 55, 60, 61	3			5, 6, 49, 54, 66, 74, 75
Social norms	The acceptable or standard/typical behaviours in groups or society, particularly related to food and eating	socio-cultural norms influencing food choice;[44] social norms around feeding behaviors;[47] food customs and fears/culinary culture, food acceptability;[54] psycho-social factors;[64] socio-cultural environment;[66] social psychology;[71] subjective norms;[76] norms of dominant society[77]	44, 47, 64, 71, 76				54, 66, 77
Types of foods available within schools and daycares	The nature and type of foods that are allowed/restricted, or that are provided (e.g., school snack programs) within daycares and schools	behavioral settings (i.e. schools, worksites); school food environment;[31] provision of healthy foods and snacks/removal of unhealthy foods from vending machines and snacks;[51] school lunch programs;[60] quality of school meals, influence of day care on child's diet;[61] organizational nutrition environment (school, work, other);[64] school cafeteria;[73] structures (school);[77] school system[78]	31, 51, 60, 61, 64, 73, 78				77
Health status	An individual's physical, mental and social well-being as well as the presence/absence of disease or infirmity	disease system;[5] disease;[19] child's health;[55] child's health problems influencing diet;[61] individual psychology, physiology;[71] HIV infection and AIDS;[75] mental health;[78] child psychological state[79]	19, 55, 61, 71, 78, 79				5, 75

Suppressed or susceptible immune system	An individual's immune system functioning at a less-than-optimal level, or in some way compromised (e.g., via HIV infection), leaving them susceptible	dispersal/displacement immunity co-infections;[3] susceptibility/immune response;[5] productive immunity;[28] HIV infection and AIDS[75]		3		28	5, 75
Age	Specific ages or age stages (e.g., elderly, newborns), particularly those that make individuals susceptible or vulnerable	host's age;[13] age;[28, 31, 40, 57, 60] ageing;[39] child's age[55]	13, 24, 31, 39, 40, 55, 57, 60			28	
Sex and gender	The biological sex and the gender of individuals	host's sex;[13] gender;[19, 40, 60, 74] sex;[31] child's sex;[55] gender differences[73]	13, 19, 31, 40, 55, 60, 73				74
Ethnicity/race	Race and ethnicity	race/ethnicity;[40; 74] race;[57] ethnicity;[60]	40, 57, 60				74
Socio-economic status	The income, social class, or standard of living of an individual/family/household, as relative to the wider community	standards of living;[2] socioeconomic conditions;[3] incomes;[5, 49, 66] cost of living;[6] maternal SES;[24] low SES education;[29] household socioeconomic status;[31] SES, family financial resources, economic-nutrition resources;[55] socioeconomic status;[60, 73, 74] family physical resources[79]	24, 29, 31, 55, 60, 73, 79	2, 3			5, 6, 49, 66, 74
Culture	Cuisine, social habits, and other characteristics of a group of people	mixed culture, effects of colonization and assimilation;[6] food culture;[32] food customs and fears/culinary culture;[54] culture;[55, 74] cultural norms and values;[65] sociocultural environment;[66, 76] cultural traditions;[66] community culture[77]	32, 55, 65, 76				6, 54, 66, 74, 77
Availability of clean, safe water	Having enough clean, safe drinking water, water to use for food preparation and processing, etc.	water filtration/treatment;[2] clean water availability;[5] drinking water;[8] clean drinking water[66]		2	8		5, 66
Caesarean birth	Caesarean versus vaginal delivery	Caesarean/Vaginal delivery;[9] birth canal vs C-section delivery;[19] C-section;[21] Caesarean birth;[25] mode and place of delivery[26]	9, 19, 21			25, 26	
Use of antibiotics	Use of antibiotics and other antimicrobials used to reduce infection	antibiotics treatment;[13] prenatal antibiotics, antibiotics during nursing, antibiotic exposure in early life, mild antibiotic disruption in early life;[19] antibiotic exposure;[21] antibiotic use;[25] early antibiotic use[26]	13, 19, 21			25, 26	

Urbanization	An increase in the population in, and growth of, cities and towns, and the related development of rural areas	Urbanisation;[1, 6, 44, 54] decreased access to land;[6] urban planning (lack of open spaces vs recreational areas for physical activity)[73]	44, 73	1			6, 54
Changes in vegetation, habitats, and ecosystems	Changes in the natural environment in an area, including vegetation cover, forests and other ecosystems; influenced by the destruction of habitats, deforestation, overfishing, etc.	deforestation, vegetation, and habitat changes of hosts;[1] vegetation/shade, behavior landscape practices;[3] soil degradation, environmental system;[5] deforestation, overfishing;[6] vegetation cover, alteration to food web structure;[7] agriculture soil and earthworms;[8] environmental health;[31] environmental change;[49] destruction of habitat and wildlife[77]	31	1, 3	7, 8		5, 6, 49, 77
Spatial co-existence of people with fauna	Spatial overlap between humans and a range of native or other animals that may serve as a food source, reservoir for infectious agents, or sources of contaminants, etc.	spatial overlap of hosts;[1] contaminant sources, reservoirs;[4] increased anthropogenic activity within Arctic, changes to bio-transport (e.g. altered migration patterns)[7]		1	4, 7		
Changes in exposure to infectious diseases	Microbial exposure and factors that change exposure, such as population programs aimed at reducing exposure to infectious diseases (e.g., vaccination; the elimination of enteropathogens like *H. pylori*)	vaccination programs, disease surveillance and monitoring;[2] elimination of enteropathogens (*H. pylori*, helminths), vaccination/reduced exposure to infectious disease;[25] exogenous microbial exposure (i.e. LPS, helminths); exposure of immune system to excess microbial products[27]		2		25, 27	
Presence of contaminants in the environment	The presence of pollution or contaminants in the local area or neighbourhood environment, either detectable by people, or not; includes the presence of contaminant sources, and their potential for food contamination	use of protective technologies (e.g. pesticides), air pollution levels, contamination pathways;[2] pollution;[5; 6; 79] import/availability of products and materials intentionally or unintentionally containing chemicals, northward shift in agriculture and chemical use, increased volume produced/consumed of emissions;[7] PFOS air/water emission;[8] environmental toxicants;[19] environmental factors (diesel exhaust, organic pollutants);[20] air/soil/water	19, 20, 79	2	7, 8, 48, 80		5, 6, 49

		contaminants;[48] environmental contaminants;[49] addition of contaminated ingredients[80]					
Early life feeding	Early life feeding patterns, including being fed formula versus breast milk, and early life nutrition	breastfeeding/formula feeding;[9] early-life nutrition;[16] nursing;[19] breastfeeding vs. formula feeding vs. early complementary feeding, infant vs. maternal control of feeding, maternal diet quality;[24] formula feeding;[25] early feedings;[26] breastfeeding;[28; 29] family feeding practices, early-life events;[31] parental feeding;[33] under/over-feeding;[38] early-life nutrition;[39] child feeding practices;[47, 60] parental feeding styles and eating behaviors;[55] food parenting practices, child's home food environment;[63] discordant feeding responsiveness;[69] feeding parenting practices[81]	9, 16, 19, 24, 29, 31, 33, 38, 39, 47, 55, 60, 63, 69, 81			25, 26, 28	
Maternal-fetal interaction	In utero experience, including maternal nutrition	healthy/LGA/SGA birth weight, maternal unhealthy dietary patterns;[9] prenatal stress (parental obesity, maternal smoking);[20] maternal-foetal interaction;[26] large birthweight, maternal nutrition, in utero growth restriction, maternal diabetes;[29] fetal programming;[35] maternal obesity, in-utero environment;[36] maternal diet and obesity, altered fetal/placental growth;[37] in-utero malnutrition[70]	9, 20, 29, 35-37, 70			26	
Globalization and increasing global trade	Worldwide interaction of cultures, economies, and politics, and increasing worldwide trade	Globalisation;[44; 54] food trade[66]	44				54, 66
Access to health care services	The ability to seek and receive appropriate or needed health services, including the inability to access services because they do not exist locally	access to health care;[2] health system;[5] health clinics with culturally competent care providers;[31] health care;[40] health care, illness management[66]	31, 40	2			5, 66
The economic environment	Society's economy, including wages, unemployment rates, wealth, resources, budget, growth, development, etc.	labor availability;[5] economic growth;[44] amount of social assistance, jobs needed;[49] national socio-economic status system/class distribution;[54] economic environment;[76] wage economy, community economic development and livelihood[77]	44, 76				5, 49, 54, 77

Food marketing and advertising	Promotion and selling of food/beverage products or services by private businesses	Marketing;[5] food labelling;[16] marketing and advertising;[29] food marketing;[32, 74] advertising;[43] media and marketing influencing child's diet;[61] consumer nutrition environment (price, promotion, placement, nutrition information), information environment (media, advertising);[64] markets;[66] advertising (no regulation vs regulation)[73]	16, 29, 32, 43, 61, 64, 73				5, 66, 74
Inter-personal influences and supports	Individual needs, pressures, expectations, assistance, and support from friends, peers, teachers, leaders, and others	community relationships;[6] peer support for healthy foods, knowledge, attitudes and beliefs about a healthy weight;[31] needs, pressures, and expectations from social networks, school, workplace, and health care providers;[32] interpersonal influences (i.e. family, peers, teachers);[40] maternal social support network;[55] peer interactions;[60] influence of child's peers;[61] psycho-social factors;[64] work/social life relationships;[65] socio-cultural environment;[66] social psychology;[71] community (social);[77] peers[78]	31, 32, 40, 55, 60, 61, 64, 65, 71, 78				6, 66, 77
Food skills and knowledge	The knowledge and abilities of individuals to prepare meals and foods, including meal planning and other factors related to self-sufficiency in feeding oneself and ones dependents	loss of traditional knowledge, loss of food-related skills;[6] education and information regarding nutrition;[16] maternal education;[24] food education;[32] parental nutrition and health knowledge; parent cognitions and beliefs (around nutrition/health);[47] dietary change, rationing, and food purchasing patterns;[49] maternal health knowledge and usage;[55] nutritional knowledge;[60] parental knowledge and beliefs about diet;[61] food procurement;[64] nutrition knowledge;[66] nutritional education[73]	16, 24, 32, 47, 55, 60, 61, 64, 73				6, 49, 66
Food production and distribution environment and infrastructure	The infrastructure and physical/agricultural processes with, and conditions under, which food is produced/harvested, stored, processed, and distributed	diet and food production;[16] food production/distribution;[54] transport and distribution, storage and processing of food, food production;[66] food production;[71] quality of harvest, storage, processing, transporting conditions and practices, insufficient or delayed drying before storage, conditions of storage[80]	16, 71		80		54, 66
Household/ family structure and dynamics	Home life, household dynamics, household structure, and family member behaviours that influence food and food behaviours	family relationships;[6] parental diet, neglect/abuse;[29] behavioral settings (i.e. home); family feeding practices, household structure, family parenting practices, family support for healthy foods;[31] needs, pressures, and expectations from family;[32] parent cognitions and beliefs (around nutrition/health), parent eating behaviors, parenting style;[47] household composition and livelihoods;[54] mother's own eating	29, 31, 32, 47, 55, 60, 61, 63-65, 78, 79, 81, 82				6, 54, 66, 77

		behaviour, maternal resources/education/intelligence/depression, parental feeding styles and eating behaviors;[55] sibling interactions, parent food preferences, parent dietary intake, parenting styles and family characteristics, types of food available in the home;[60] parental motivation to encourage healthy foods, parental control of child's diet, parental role modelling of healthy diet, parental use of food to manipulate child's behavior, importance of eating together as a family, parental guilt and emotions regarding child's diet, family views influencing diet, parental perceptions about lack of time for child's healthy diet;[61] food parenting practices, parental dietary behavior, child's home food environment, parenting context;[63] nutrition environment (home);[64] home relationships;[65] intra-household distribution of food;[66] household (gendered division of labor, power relations, decision-making);[77] family;[78] family social environment (violent, parent psychological state, parenting);[79] feeding parenting practices;[81] family system variables (competence, satisfaction, warmth, cohesion), parenting style[82]					
Built environment	The overall human-made spaces, including land use, infrastructure	decreased access to land, physical environment;[6] built environment;[16, 29, 78] changes to built environment;[44] built environment/infrastructure;[49] neighborhood, use of public spaces;[65] behavioral settings (i.e. neighborhoods); urban planning (lack of open spaces vs recreational areas for physical activity);[73] physical environment;[76] community (physical infrastructure, services)[77]	16, 29, 44, 65, 73, 76, 78				6, 49, 77
Agricultural intensification	An increase in agricultural production per unit of inputs (which may be labour, land, time, fertilizer, seed, feed or cash)	agricultural intensification;[1] northward shift in agriculture and chemical use[7]		1	7		

The food supply	The amount and types of food being produced, including the vulnerability (resilience) of the supply to interruptions or perturbations, and its sustainability	sustainability of food production, agri-food system;[5] alteration to food web structure;[7] stability (in food availability, food access, food utilization);[66] food supply;[74] vulnerability context (shocks, trends, socio-ecological systems, etc)[77]			7		5, 66, 74, 77
Population demographics	Population characteristics, especially those regarding average age, income, education, race, sex, etc.	population growth and demographic change;[2] community, demographic, and societal characteristics[60]	60	2			
Community dynamics and well-being	Characteristics, resources (including social capital), and dynamics within neighbourhoods and communities	community self- and determination;[31] community capacity and engagement, community-level initiatives;[49] community, demographic, and societal characteristics, crime rates and neighborhood safety;[60] community (social);[77] neighborhood resources, neighborhood social environment (violence, social capital)[79]	31, 60, 79				49, 77
Time and resources needed to eat 'healthy'	Time and resource 'costs' of healthy eating	time and money for food;[32] less time for hunting/fishing/cooking traditional food due to other employment;[49] economic-nutrition resources;[55] increased opportunity to eat;[56, 83] parental perceptions about lack of time for child's healthy diet;[61] increased time to eat[62; 67]	32, 55, 56, 61, 62, 67, 83				49
Government and industry laws, policies and regulations	Processes used to monitor and enforce laws	government restrictions;[6] policies relating to food;[31] food policies and regulations;[32] government and policy;[40] government and industry policies;[64] policies and institutions;[66] advertising (no regulation vs regulation);[73] political environment;[76] regulation, structures (levels of government, laws); community politics, policies;[77] healthy policy environment;[78] existing regulation regarding OTA concentrations in cereal foodstuffs[80]	31, 32, 40, 64, 73, 76, 78		80		6, 66, 77
**Infectious Foodborne Illness**	Infections that are commonly or can be transmitted via food	adenovirus infection;[72] soil-transmitted helminth infection[3]	72	3			

**Dietary Contaminants**	The presence in food of harmful chemicals, toxicants, or pollutants which have the potential to cause consumer illness	contamination of foods[6]					6
**Food Insecurity**	The state of being without reliable access to a sufficient quantity of affordable, nutritious food	food insecurity;[29] household food security[31]	29, 31				

References

1. Atkinson JA, Gray DJ, Clements AC, Barnes TS, McManus DP, Yang YR. Environmental changes impacting Echinococcus transmission: research to support predictive surveillance and control. *Glob Chang Biol.* 2013; 19(3): 677-88.

2. Patz JA, Hahn MB. Climate change and human health: a One Health approach. *Curr Top Microbiol Immunol*. 2013; 366: 141-71.

3. Weaver HJ, Hawdon JM, Hoberg EP. Soil-transmitted helminthiases: implications of climate change and human behavior. *Trends Parasitol.* 2010; 26(12): 574-81.

4. Ciaccio CE, Kennedy K, Portnoy JM. A new model for environmental assessment and exposure reduction. *Curr Allergy Asthma Rep*. 2012; 12(6): 650-5.

5. Hammond RA, Dube L. A systems science perspective and transdisciplinary models for food and nutrition security. *P Natl A Sci.* 2012; 109(31): 12356.

6. Elliott B, Jayatilaka D, Brown C, Varley L, Corbett KK. "We are not being heard": Aboriginal perspectives on traditional foods access and food security. *J Environ Public Health*. 2012; 2012(130945).

7. Armitage JM, Quinn CL, Wania F. Global climate change and contaminants--an overview of opportunities and priorities for modelling the potential implications for long-term human exposure to organic compounds in the Arctic. *J Environ Monit*. 2011; 13(6): 1532-46.

8. Brambilla G, D'Hollander W, Oliaei F, Stahl T, Weber R. Pathways and factors for food safety and food security at PFOS contaminated sites within a problem based learning approach. *Chemosphere*. 2014.

9. Paliy O, Piyathilake CJ, Kozyrskyj A, Celep G, Marotta F, Rastmanesh R. Excess body weight during pregnancy and offspring obesity: potential mechanisms. *Nutrition.* 2014; 30(3): 245-51.

10. Pataky Z, Bobbioni-Harsch E, Golay A. Obesity: a complex growing challenge. *Exp Clin Endocrinol Diabetes.* 2010; 118(7): 427-33.

11. Cox AJ, West NP, Cripps AW. Obesity, inflammation, and the gut microbiota. *Lancet Diabetes Endocrinol*. 2015; 3(3): 207-15.

12. Teixeira TF, Collado MC, Ferreira CL, Bressan J, Peluzio Mdo C. Potential mechanisms for the emerging link between obesity and increased intestinal permeability. *Nutr Res.* 2012; 32(9): 637-47.

13. Delzenne NM, Neyrinck AM, Backhed F, Cani PD. Targeting gut microbiota in obesity: effects of prebiotics and probiotics. *Nat Rev Endocrinol*. 2011; 7(11): 639-46.

14. Moran CP, Shanahan F. Gut microbiota and obesity: role in aetiology and potential therapeutic target. *Best Pract Res Clin Gastroenterol.* 2014; 28(4): 585-97.

15. Ganu RS, Harris RA, Collins K, Aagaard KM. Maternal diet: a modulator for epigenomic regulation during development in nonhuman primates and humans. *Int J Obes Suppl.* 2012; 2(Suppl 2): S14-8.

16. Palou A, Bonet ML. Challenges in obesity research. *Nutr Hosp.* 2013; 28 Suppl 5: 144-53.

17. Flint HJ. Obesity and the gut microbiota. *J Clin Gastroenterol.* 2011; 45 Suppl: S128-32.

18. Tsai YT, Cheng PC, Pan TM. Anti-obesity effects of gut microbiota are associated with lactic acid bacteria. *Appl Microbiol Biotechnol.* 2014; 98(1): 1-10.

19. Cox LM, Blaser MJ. Antibiotics in early life and obesity. *Nat Rev Endocrinol*. 2015; 11(3): 182-90.

20. Holvoet P. Stress in obesity and associated metabolic and cardiovascular disorders. *Scientifica* (Cairo). 2012; 2012: 205027.

21. Cox LM, Blaser MJ. Pathways in microbe-induced obesity. *Cell Metab*. 2013; 17(6): 883-94.

22. Prescott SL. Early-life environmental determinants of allergic diseases and the wider pandemic of inflammatory noncommunicable diseases. *J Allergy Clin Immunol.* 2013; 131(1): 23-30.

23. Burcelin R. Regulation of metabolism: a cross talk between gut microbiota and its human host. *Physiology (Bethesda).* 2012; 27(5): 300-7.

24. Thompson AL. Developmental origins of obesity: early feeding environments, infant growth, and the intestinal microbiome. *Am J Hum Biol.* 2012; 24(3): 350-60.

25. Feehley T, Stefka AT, Cao S, Nagler CR. Microbial regulation of allergic responses to food. *Semin Immunopathol.* 2012; 34(5): 671-88.

26. Tsabouri S, Priftis KN, Chaliasos N, Siamopoulou A. Modulation of gut microbiota downregulates the development of food allergy in infancy. *Allergol Immunopathol* (Madr). 2014; 42(1): 69-77.

27. Vassallo MF, Camargo CA,Jr. Potential mechanisms for the hypothesized link between sunshine, vitamin D, and food allergy in children. *J Allergy Clin Immunol.* 2010; 126(2): 217-22.

28. Brandtzaeg P. Food allergy: separating the science from the mythology. *Nat Rev Gastroenterol Hepatol.* 2010; 7(7): 380-400.

29. Monasta L, Batty GD, Cattaneo A, Lutje V, Ronfani L, Van Lenthe FJ, et al. Early-life determinants of overweight and obesity: a review of systematic reviews. *Obes Rev.* 2010; 11(10): 695-708.

30. deShazo RD, Hall JE, Skipworth LB. Obesity Bias, Medical Technology, and the Hormonal Hypothesis: Should We Stop Demonizing Fat People? *Am J Med*. 2014.

31. Willows ND, Hanley AJ, Delormier T. A socioecological framework to understand weight-related issues in Aboriginal children in Canada. *Appl Physiol Nutr Metab*. 2012; 37(1): 1-13.

32. Lunn TE, Nowson CA, Worsley A, Torres SJ. Does personality affect dietary intake? *Nutrition.* 2014; 30(4): 403-9.

33. Carnell S, Kim Y, Pryor K. Fat brains, greedy genes, and parent power: a biobehavioural risk model of child and adult obesity. *Int Rev Psychiatry*. 2012; 24(3): 189-99.

34. Carnell S, Gibson C, Benson L, Ochner CN, Geliebter A. Neuroimaging and obesity: current knowledge and future directions. *Obes Rev*. 2012; 13(1): 43-56.

35. Tounian P. Programming towards childhood obesity. *Ann Nutr Metab.* 2011; 58 Suppl 2: 30-41.

36. Galliano D, Bellver J. Female obesity: short- and long-term consequences on the offspring. *Gynecol Endocrinol.* 2013; 29(7): 626-31.

37. O'Reilly JR, Reynolds RM. The risk of maternal obesity to the long-term health of the offspring. *Clin Endocrinol* (Oxf). 2013; 78(1): 9-16.

38. Bourguignon JP, Parent AS. Early homeostatic disturbances of human growth and maturation by endocrine disrupters. *Curr Opin Pediatr*. 2010; 22(4): 470-7.

39. Zheng J, Xiao X, Zhang Q, Yu M. DNA methylation: the pivotal interaction between early-life nutrition and glucose metabolism in later life. *Br J Nutr.* 2014; 112(11): 1850-7.

40. Wang ML, Peterson KE, McCormick MC, Austin SB. Environmental factors associated with disordered weight-control behaviours among youth: a systematic review. *Public Health Nutr.* 2014; 17(7): 1654-67.

41. Davidson TL, Sample CH, Swithers SE. An application of Pavlovian principles to the problems of obesity and cognitive decline. *Neurobiol Learn Mem*. 2014; 108: 172-84.

42. Kanoski SE, Davidson TL. Western diet consumption and cognitive impairment: links to hippocampal dysfunction and obesity. *Physiol Behav.* 2011; 103(1): 59-68.

43. Rolls ET. Taste, olfactory and food texture reward processing in the brain and obesity. *Int J Obes* (Lond). 2011; 35(4): 550-61.

44. Malik VS, Willett WC, Hu FB. Global obesity: trends, risk factors and policy implications. *Nat Rev Endocrinol.* 2013; 9(1): 13-27.

45. Wells JC, Siervo M. Obesity and energy balance: is the tail wagging the dog? *Eur J Clin Nutr.* 2011; 65(11): 1173-89.

46. Ryan KK, Woods SC, Seeley RJ. Central nervous system mechanisms linking the consumption of palatable high-fat diets to the defense of greater adiposity. *Cell Metab.* 2012; 15(2): 137-49.

47. Skouteris H, McCabe M, Swinburn B, Newgreen V, Sacher P, Chadwick P. Parental influence and obesity prevention in pre-schoolers: a systematic review of interventions. *Obes Rev.* 2011; 12(5): 315-28.

48. Lassiter MG, Owens EO, Patel MM, Kirrane E, Madden M, Richmond-Bryant J, et al. Cross-species coherence in effects and modes of action in support of causality determinations in the U.S. Environmental Protection Agency's Integrated Science Assessment for Lead. *Toxicology.* 2015; 330: 19-40.

49. Skinner K, Hanning RM, Desjardins E, Tsuji L. Giving voice to food insecurity in a remote indigenous community in subarctic Ontario, Canada: traditional ways, ways to cope, ways forward. *BMC Public Health.* 2013; 13: 427.

50. Soares MJ, Pathak K, Calton EK. Calcium and vitamin D in the regulation of energy balance: where do we stand? *Int J Mol Sci.* 2014; 15(3): 4938-45.

51. Brennan LK, Brownson RC, Orleans CT. Childhood obesity policy research and practice: evidence for policy and environmental strategies. *Am J Prev Med*. 2014; 46(1): e1-16.

52. Fardet A. New hypotheses for the health-protective mechanisms of whole-grain cereals: what is beyond fibre? *Nutr Res Rev*. 2010; 23(1): 65-134.

53. Mangge H, Summers K, Almer G, Prassl R, Weghuber D, Schnedl W, et al. Antioxidant food supplements and obesity-related inflammation. *Curr Med Chem.* 2013; 20(18): 2330-7.

54. Dixon J, Omwega AM, Friel S, Burns C, Donati K, Carlisle R. The health equity dimensions of urban food systems. *J Urban Health.* 2007; 84(1).

55. El-Behadli AF, Sharp C, Hughes SO, Obasi EM, Nicklas TA. Maternal depression, stress and feeding styles: towards a framework for theory and research in child obesity. *Br J Nutr.* 2015; 113 Suppl: S55-71.

56. Hart CN, Cairns A, Jelalian E. Sleep and obesity in children and adolescents. *Pediatr Clin North Am.* 2011; 58(3): 715-33.

57. Melzer K, Schutz Y. Pre-pregnancy and pregnancy predictors of obesity. *Int J Obes (Lond).* 2010; 34 Suppl 2: S44-52.

58. Sinha R, Jastreboff AM. Stress as a common risk factor for obesity and addiction. *Biol Psychiatry.* 2013; 73(9): 827-35.

59. Singh M. Mood, food, and obesity. *Front Psychol.* 2014; 5: 925.

60. Galvez MP, Pearl M, Yen IH. Childhood obesity and the built environment. *Curr Opin Pediatr.* 2010; 22(2): 202-7.

61. Pocock M, Trivedi D, Wills W, Bunn F, Magnusson J. Parental perceptions regarding healthy behaviours for preventing overweight and obesity in young children: a systematic review of qualitative studies. *Obes Rev.* 2010; 11(5): 338-53.

62. Shlisky JD, Hartman TJ, Kris-Etherton PM, Rogers CJ, Sharkey NA, Nickols-Richardson SM. Partial sleep deprivation and energy balance in adults: an emerging issue for consideration by dietetics practitioners. *J Acad Nutr Diet.* 2012; 112(11): 1785-97.

63. Larsen JK, Hermans RC, Sleddens EF, Engels RC, Fisher JO, Kremers SS. How parental dietary behavior and food parenting practices affect children's dietary behavior. Interacting sources of influence? *Appetite.* 2015; 89: 246-57.

64. Minaker LM. *Evaluating food environment assessment methodologies: a multi-level examination of associations between food environments and individual outcomes* [PhD thesis]. Edmonton, AB: University of Alberta; 2013.

65. Perez-Escamilla R, Kac G. Childhood obesity prevention: a life-course framework. *Int J Obes* Suppl. 2013; 3(Suppl 1): S3-5.

66. Burchi F, Fanzo J, Frison E. The role of food and nutrition system approaches in tackling hidden hunger. *Int J Environ Res Public Health*. 2011; 8(2): 358-73.

67. Knutson KL. Does inadequate sleep play a role in vulnerability to obesity? *Am J Hum Biol.* 2012; 24(3): 361-71.

68. Bray GA, Popkin BM. Calorie-sweetened beverages and fructose: what have we learned 10 years later. *Pediatr Obes*. 2013; 8(4): 242-8.

69. DiSantis KI, Hodges EA, Johnson SL, Fisher JO. The role of responsive feeding in overweight during infancy and toddlerhood: a systematic review. *Int J Obes (Lond).* 2011; 35(4): 480-92.

70. Calkins K, Devaskar SU. Fetal origins of adult disease. *Curr Probl Pediatr Adolesc Health Care*. 2011; 41(6): 158-76.

71. Finegood DT, Merth TDN, Rutter H. Implications of the Foresight Obesity System Map for Solutions to Childhood Obesity. *Obesity*. 2010; 18(S1): S13,S14-S16.

72. Hur SJ, Kim DH, Chun SC, Lee SK. Effect of adenovirus and influenza virus infection on obesity. *Life Sci.* 2013; 93(16): 531-5.

73. Franco M, Sanz B, Otero L, Dominguez-Vila A, Caballero B. Prevention of childhood obesity in Spain: a focus on policies outside the health sector. SESPAS report 2010. *Gac Sanit.* 2010; 24 Suppl 1: 49-55.

74. Neff RA, Palmer AM, McKenzie SE, Lawrence RS. Food systems and public health disparities. *J Hunger Environ Nutr*. 2009; 4: 282.

75. Frega R, Duffy F, Rawat R, Grede N. Food insecurity in the context of HIV/AIDS: a framework for a new era of programming. *Food Nutr Bull.* 2010; 31(4): S292-312.

76. Kremers SP. Theory and practice in the study of influences on energy balance-related behaviors. *Patient Educ Couns*. 2010; 79(3): 291-8.

77. Thompson S, Kamal AG. Community development to feed the family in northern Manitoba communities: Evaluating food activities on their food sovereignty, food security, and sustainable livelihood outcomes. *C J Nonprofit Soc Econ Res.* 2012; 3(2): 43,44-66.

78. Cockrell Skinner A, Foster EM. Systems science and childhood obesity: a systematic review and new directions. *J Obes*. 2013; 2013: 129193.

79. Schreier HM, Chen E. Socioeconomic status and the health of youth: a multilevel, multidomain approach to conceptualizing pathways. *Psychol Bull.* 2013; 139(3): 606-54.

80. Duarte SC, Pena A, Lino CM. A review on ochratoxin A occurrence and effects of processing of cereal and cereal derived food products. *Food Microbiol*. 2010; 27(2): 187-98.

81. Faith MS, Van Horn L, Appel LJ, Burke LE, Carson JA, Franch HA, et al. Evaluating parents and adult caregivers as "agents of change" for treating obese children: evidence for parent behavior change strategies and research gaps: a scientific statement from the American Heart Association. *Circulation*. 2012; 125(9): 1186-207.

82. Kitzman-Ulrich H, Wilson DK, St George SM, Lawman H, Segal M, Fairchild A. The integration of a family systems approach for understanding youth obesity, physical activity, and dietary programs. *Clin Child Fam Psychol Rev.* 2010; 13(3): 231-53.

83. Zimberg IZ, Damaso A, Del Re M, Carneiro AM, de Sa Souza H, de Lira FS, et al. Short sleep duration and obesity: mechanisms and future perspectives. *Cell Biochem Funct*. 2012; 30(6): 524-9.
