# Supplementary material for: Food, health, and complexity: towards a conceptual understanding to guide collaborative public health action
Source: BMC Public Health. 2016 Jun 8;16:487. doi: 10.1186/s12889-016-3142-6 (PMC4898364; doi:10.1186/s12889-016-3142-6)

Supplemental Online Appendix B. The 49 Drivers, Common to Two or More of the Five Population Health Issues Related to Food, as Identified from the Literature; Dashed Arrows are Interconnections Between Drivers, and Coloured Arrows are Direct Connections to a Given Population Health Issue.

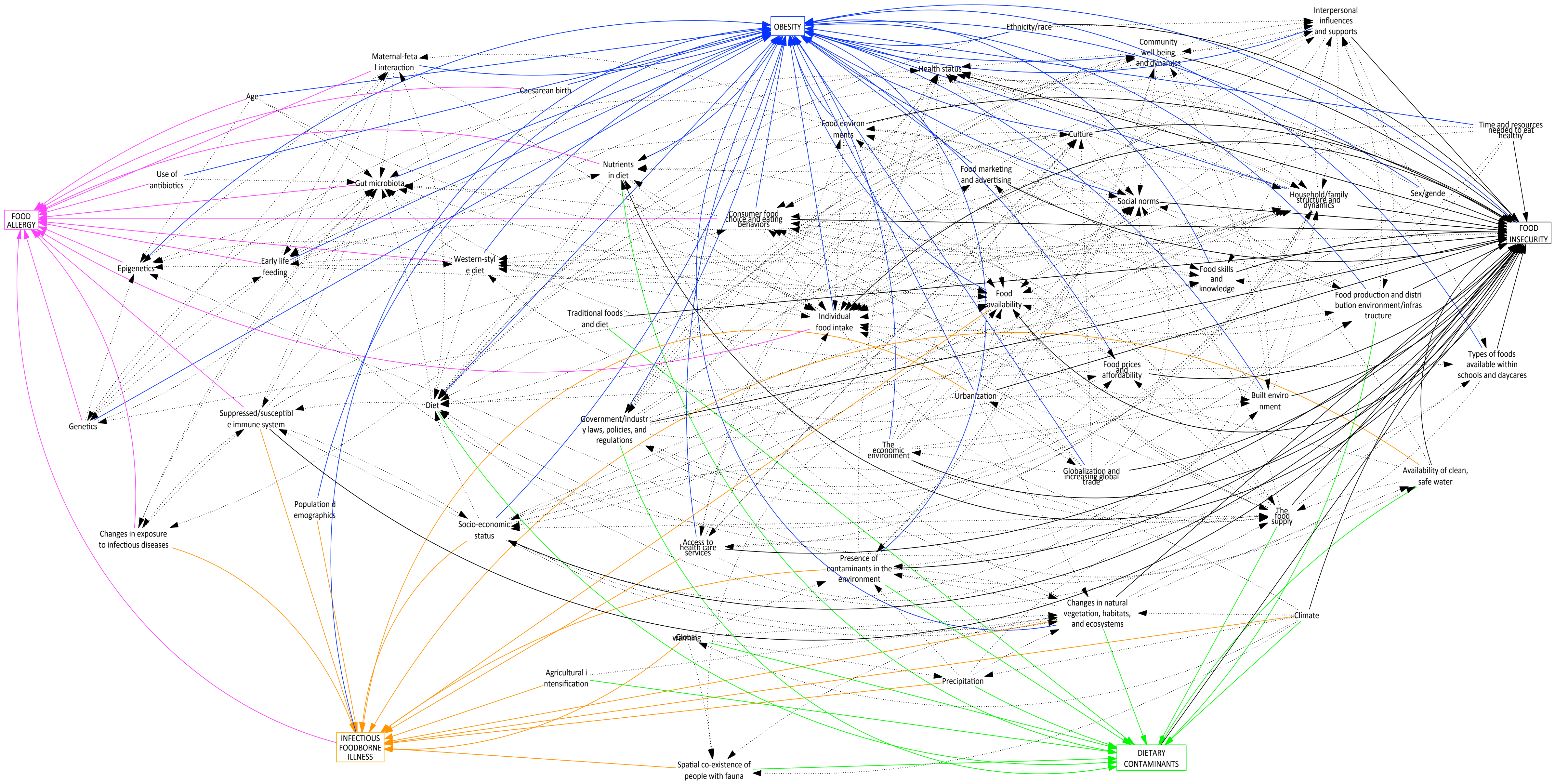

Supplement: Additional file 2: — The 49 drivers, common to two or more of the five population health issues related to food, as identified from the literature; dashed arrows are interconnections between drivers, and coloured arrows are direct connections to a given population health issue. (PDF 86 kb) [file 12889_2016_3142_MOESM2_ESM.pdf]
