# Supplementary material for: Food, health, and complexity: towards a conceptual understanding to guide collaborative public health action
Source: BMC Public Health. 2016 Jun 8;16:487. doi: 10.1186/s12889-016-3142-6 (PMC4898364; doi:10.1186/s12889-016-3142-6)
Supplement: Additional file 3: — Tree diagrams showing the relevant drivers for each of the five population health issues related to food; drivers in brackets are those that are also found elsewhere in the particular tree. (PDF 207 kb) [file 12889_2016_3142_MOESM3_ESM.pdf]

Supplemental Online Appendix C. Tree Diagrams Showing the Relevant Drivers for Each of the Five Population Health Issues Related to Food; Drivers in Brackets are Those that are Also Found Elsewhere in the Particular Tree

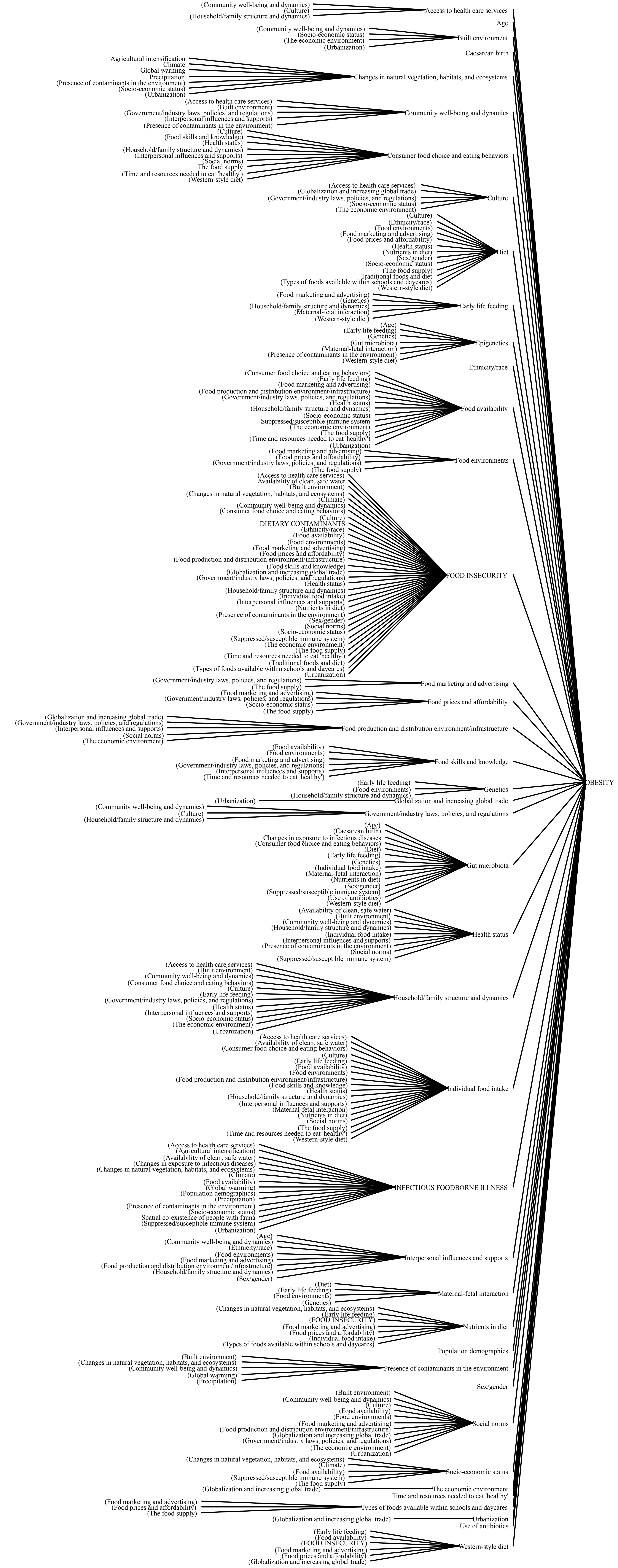

(Community well-being and dynamics)
(Culture)
(Household/family structure and dynamics)
Access to health care services
Age
(Community well-being and dynamics)
(Socio-economic status)
(The economic environment)
(Urbanization)
Built environment
Caesarean birth
Agricultural intensification
Climate
Global warming
Precipitation
(Presence of contaminants in the environment)
(Socio-economic status)
(Urbanization)
Changes in natural vegetation, habitats, and ecosystems
(Access to health care services)
(Built environment)
(Government/industry laws, policies, and regulations)
(Interpersonal influences and supports)
(Presence of contaminants in the environment)
Community well-being and dynamics
(Culture)
(Food skills and knowledge)
(Health status)
(Household/family structure and dynamics)
(Interpersonal influences and supports)
(Social norms)
The food supply
(Time and resources needed to eat 'healthy')
(Western-style diet)
Consumer food choice and eating behaviors
(Access to health care services)
(Globalization and increasing global trade)
(Government/industry laws, policies, and regulations)
(Socio-economic status)
(The economic environment)
Culture
(Culture)
(Ethnicity/race)
(Food environments)
(Food marketing and advertising)
(Food prices and affordability)
(Health status)
(Nutrients in diet)
(Sex/gender)
(Socio-economic status)
(The food supply)
Traditional foods and diet
(Types of foods available within schools and daycares)
(Western-style diet)
Diet
(Food marketing and advertising)
(Genetics)
(Household/family structure and dynamics)
(Maternal-fetal interaction)
(Western-style diet)
Early life feeding
(Age)
(Early life feeding)
(Genetics)
(Gut microbiota)
(Maternal-fetal interaction)
(Presence of contaminants in the environment)
(Western-style diet)
Epigenetics
Ethnicity/race
(Consumer food choice and eating behaviors)
(Early life feeding)
(Food marketing and advertising)
(Food production and distribution environment/infrastructure)
(Government/industry laws, policies, and regulations)
(Health status)
(Household/family structure and dynamics)
(Socio-economic status)
Suppressed/susceptible immune system
(The economic environment)
(The food supply)
(Time and resources needed to eat 'healthy')
(Urbanization)
Food availability
(Food marketing and advertising)
(Food prices and affordability)
(Government/industry laws, policies, and regulations)
(The food supply)
Food environments
(Access to health care services)
Availability of clean, safe water
(Built environment)
(Changes in natural vegetation, habitats, and ecosystems)
(Climate)
(Community well-being and dynamics)
(Consumer food choice and eating behaviors)
(Culture)
DIETARY CONTAMINANTS
(Ethnicity/race)
(Food availability)
(Food environments)
(Food marketing and advertising)
(Food prices and affordability)
(Food production and distribution environment/infrastructure)
(Food skills and knowledge)
(Globalization and increasing global trade)
(Government/industry laws, policies, and regulations)
(Health status)
(Household/family structure and dynamics)
(Individual food intake)
(Interpersonal influences and supports)
(Nutrients in diet)
(Presence of contaminants in the environment)
(Sex/gender)
(Social norms)
(Socio-economic status)
(Suppressed/susceptible immune system)
(The economic environment)
(The food supply)
(Time and resources needed to eat 'healthy')
(Traditional foods and diet)
(Types of foods available within schools and daycares)
(Urbanization)
FOOD INSECURITY
(Government/industry laws, policies, and regulations)
(The food supply)
Food marketing and advertising
(Food marketing and advertising)
(Government/industry laws, policies, and regulations)
(Socio-economic status)
(The food supply)
Food prices and affordability
(Globalization and increasing global trade)
(Government/industry laws, policies, and regulations)
(Interpersonal influences and supports)
(Social norms)
(The economic environment)
Food production and distribution environment/infrastructure
(Food availability)
(Food environments)
(Food marketing and advertising)
(Government/industry laws, policies, and regulations)
(Interpersonal influences and supports)
(Time and resources needed to eat 'healthy')
Food skills and knowledge
(Early life feeding)
(Food environments)
(Household/family structure and dynamics)
Genetics
(Urbanization)
Globalization and increasing global trade
(Community well-being and dynamics)
(Culture)
(Household/family structure and dynamics)
Government/industry laws, policies, and regulations
OBESITY
(Age)
(Caesarean birth)
Changes in exposure to infectious diseases
(Consumer food choice and eating behaviors)
(Diet)
(Early life feeding)
(Genetics)
(Individual food intake)
(Maternal-fetal interaction)
(Nutrients in diet)
(Sex/gender)
(Suppressed/susceptible immune system)
(Use of antibiotics)
(Western-style diet)
Gut microbiota
(Availability of clean, safe water)
(Built environment)
(Community well-being and dynamics)
(Household/family structure and dynamics)
(Individual food intake)
(Interpersonal influences and supports)
(Presence of contaminants in the environment)
(Social norms)
(Suppressed/susceptible immune system)
Health status
(Access to health care services)
(Built environment)
(Community well-being and dynamics)
(Consumer food choice and eating behaviors)
(Culture)
(Early life feeding)
(Government/industry laws, policies, and regulations)
(Health status)
(Interpersonal influences and supports)
(Socio-economic status)
(The economic environment)
(Urbanization)
Household/family structure and dynamics
(Access to health care services)
(Availability of clean, safe water)
(Consumer food choice and eating behaviors)
(Culture)
(Early life feeding)
(Food availability)
(Food environments)
(Food production and distribution environment/infrastructure)
(Food skills and knowledge)
(Health status)
(Household/family structure and dynamics)
(Interpersonal influences and supports)
(Maternal-fetal interaction)
(Nutrients in diet)
(Social norms)
(The food supply)
(Time and resources needed to eat 'healthy')
(Western-style diet)
Individual food intake
(Access to health care services)
(Agricultural intensification)
(Availability of clean, safe water)
(Changes in exposure to infectious diseases)
(Changes in natural vegetation, habitats, and ecosystems)
(Climate)
(Food availability)
(Global warming)
(Population demographics)
(Precipitation)
(Presence of contaminants in the environment)
(Socio-economic status)
Spatial co-existence of people with fauna
(Suppressed/susceptible immune system)
(Urbanization)
INFECTIOUS FOODBORNE ILLNESS
(Age)
(Community well-being and dynamics)
(Ethnicity/race)
(Food environments)
(Food marketing and advertising)
(Food production and distribution environment/infrastructure)
(Household/family structure and dynamics)
(Sex/gender)
Interpersonal influences and supports
(Diet)
(Early life feeding)
(Food environments)
(Genetics)
Maternal-fetal interaction
(Changes in natural vegetation, habitats, and ecosystems)
(Early life feeding)
(FOOD INSECURITY)
(Food marketing and advertising)
(Food prices and affordability)
(Individual food intake)
(Types of foods available within schools and daycares)
Nutrients in diet
Population demographics
(Built environment)
(Changes in natural vegetation, habitats, and ecosystems)
(Community well-being and dynamics)
(Global warming)
(Precipitation)
Presence of contaminants in the environment
Sex/gender
(Built environment)
(Community well-being and dynamics)
(Culture)
(Food availability)
(Food environments)
(Food marketing and advertising)
(Food production and distribution environment/infrastructure)
(Globalization and increasing global trade)
(Government/industry laws, policies, and regulations)
(The economic environment)
(Urbanization)
Social norms
(Changes in natural vegetation, habitats, and ecosystems)
(Climate)
(Food availability)
(Suppressed/susceptible immune system)
(The food supply)
Socio-economic status
(Globalization and increasing global trade)
The economic environment
Time and resources needed to eat 'healthy'
(Food marketing and advertising)
(Food prices and affordability)
(The food supply)
Types of foods available within schools and daycares
(Globalization and increasing global trade)
Urbanization
Use of antibiotics
(Early life feeding)
(Food availability)
(FOOD INSECURITY)
(Food marketing and advertising)
(Food prices and affordability)
(Globalization and increasing global trade)
Western-style diet

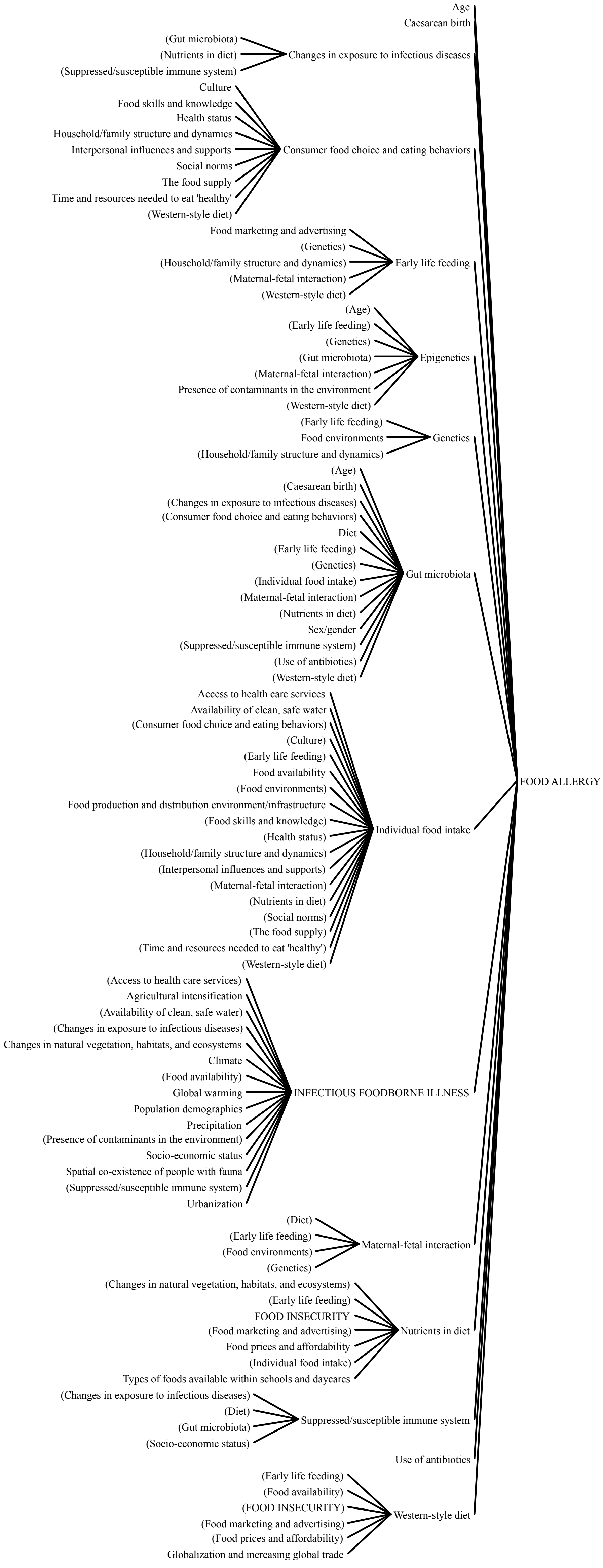

Age
Caesarean birth
(Gut microbiota)
(Nutrients in diet)
(Suppressed/susceptible immune system)
Changes in exposure to infectious diseases
Culture
Food skills and knowledge
Health status
Household/family structure and dynamics
Interpersonal influences and supports
Social norms
The food supply
Time and resources needed to eat 'healthy'
(Western-style diet)
Consumer food choice and eating behaviors
Food marketing and advertising
(Genetics)
(Household/family structure and dynamics)
(Maternal-fetal interaction)
(Western-style diet)
Early life feeding
(Age)
(Early life feeding)
(Genetics)
(Gut microbiota)
(Maternal-fetal interaction)
Presence of contaminants in the environment
(Western-style diet)
Epigenetics
(Early life feeding)
Food environments
(Household/family structure and dynamics)
Genetics
(Age)
(Caesarean birth)
(Changes in exposure to infectious diseases)
(Consumer food choice and eating behaviors)
Diet
(Early life feeding)
(Genetics)
(Individual food intake)
(Maternal-fetal interaction)
(Nutrients in diet)
Sex/gender
(Suppressed/susceptible immune system)
(Use of antibiotics)
(Western-style diet)
Gut microbiota
Access to health care services
Availability of clean, safe water
(Consumer food choice and eating behaviors)
(Culture)
(Early life feeding)
Food availability
(Food environments)
Food production and distribution environment/infrastructure
(Food skills and knowledge)
(Health status)
(Household/family structure and dynamics)
(Interpersonal influences and supports)
(Maternal-fetal interaction)
(Nutrients in diet)
(Social norms)
(The food supply)
(Time and resources needed to eat 'healthy')
(Western-style diet)
Individual food intake
FOOD ALLERGY
(Access to health care services)
Agricultural intensification
(Availability of clean, safe water)
(Changes in exposure to infectious diseases)
Changes in natural vegetation, habitats, and ecosystems
Climate
(Food availability)
Global warming
Population demographics
Precipitation
(Presence of contaminants in the environment)
Socio-economic status
Spatial co-existence of people with fauna
(Suppressed/susceptible immune system)
Urbanization
INFECTIOUS FOODBORNE ILLNESS
(Diet)
(Early life feeding)
(Food environments)
(Genetics)
Maternal-fetal interaction
(Changes in natural vegetation, habitats, and ecosystems)
(Early life feeding)
FOOD INSECURITY
(Food marketing and advertising)
Food prices and affordability
(Individual food intake)
Types of foods available within schools and daycares
Nutrients in diet
(Changes in exposure to infectious diseases)
(Diet)
(Gut microbiota)
(Socio-economic status)
Suppressed/susceptible immune system
Use of antibiotics
(Early life feeding)
(Food availability)
(FOOD INSECURITY)
(Food marketing and advertising)
(Food prices and affordability)
Globalization and increasing global trade
Western-style diet

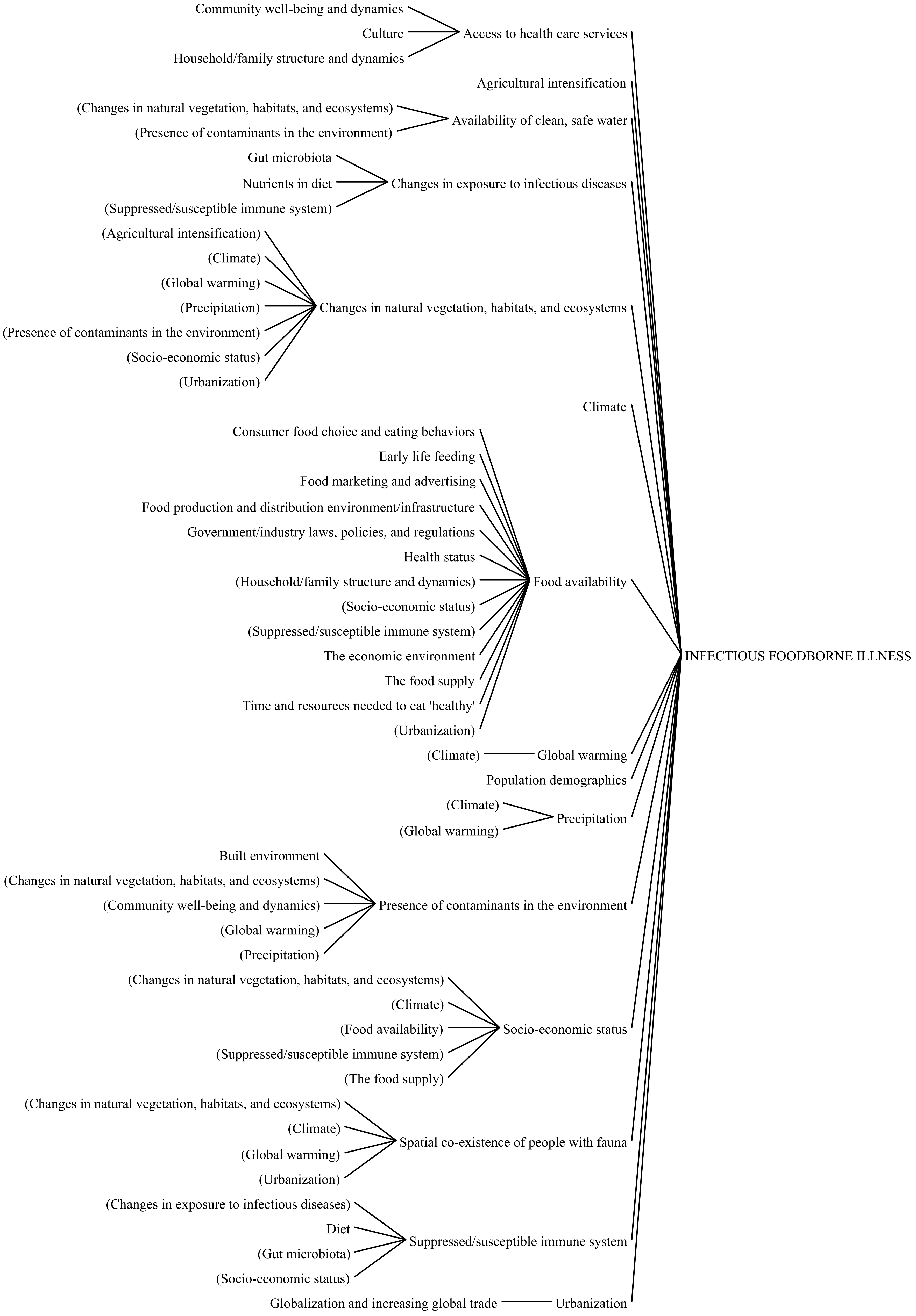

Community well-being and dynamics
Culture
Household/family structure and dynamics
Access to health care services
Agricultural intensification
(Changes in natural vegetation, habitats, and ecosystems)
(Presence of contaminants in the environment)
Availability of clean, safe water
Gut microbiota
Nutrients in diet
(Suppressed/susceptible immune system)
Changes in exposure to infectious diseases
(Agricultural intensification)
(Climate)
(Global warming)
(Precipitation)
(Presence of contaminants in the environment)
(Socio-economic status)
(Urbanization)
Changes in natural vegetation, habitats, and ecosystems
Climate
Consumer food choice and eating behaviors
Early life feeding
Food marketing and advertising
Food production and distribution environment/infrastructure
Government/industry laws, policies, and regulations
Health status
(Household/family structure and dynamics)
(Socio-economic status)
(Suppressed/susceptible immune system)
The economic environment
The food supply
Time and resources needed to eat 'healthy'
(Urbanization)
Food availability
INFECTIOUS FOODBORNE ILLNESS
(Climate)
Global warming
Population demographics
(Climate)
(Global warming)
Precipitation
Built environment
(Changes in natural vegetation, habitats, and ecosystems)
(Community well-being and dynamics)
(Global warming)
(Precipitation)
Presence of contaminants in the environment
(Changes in natural vegetation, habitats, and ecosystems)
(Climate)
(Food availability)
(Suppressed/susceptible immune system)
(The food supply)
Socio-economic status
(Changes in natural vegetation, habitats, and ecosystems)
(Climate)
(Global warming)
(Urbanization)
Spatial co-existence of people with fauna
(Changes in exposure to infectious diseases)
Diet
(Gut microbiota)
(Socio-economic status)
Suppressed/susceptible immune system
Globalization and increasing global trade
Urbanization

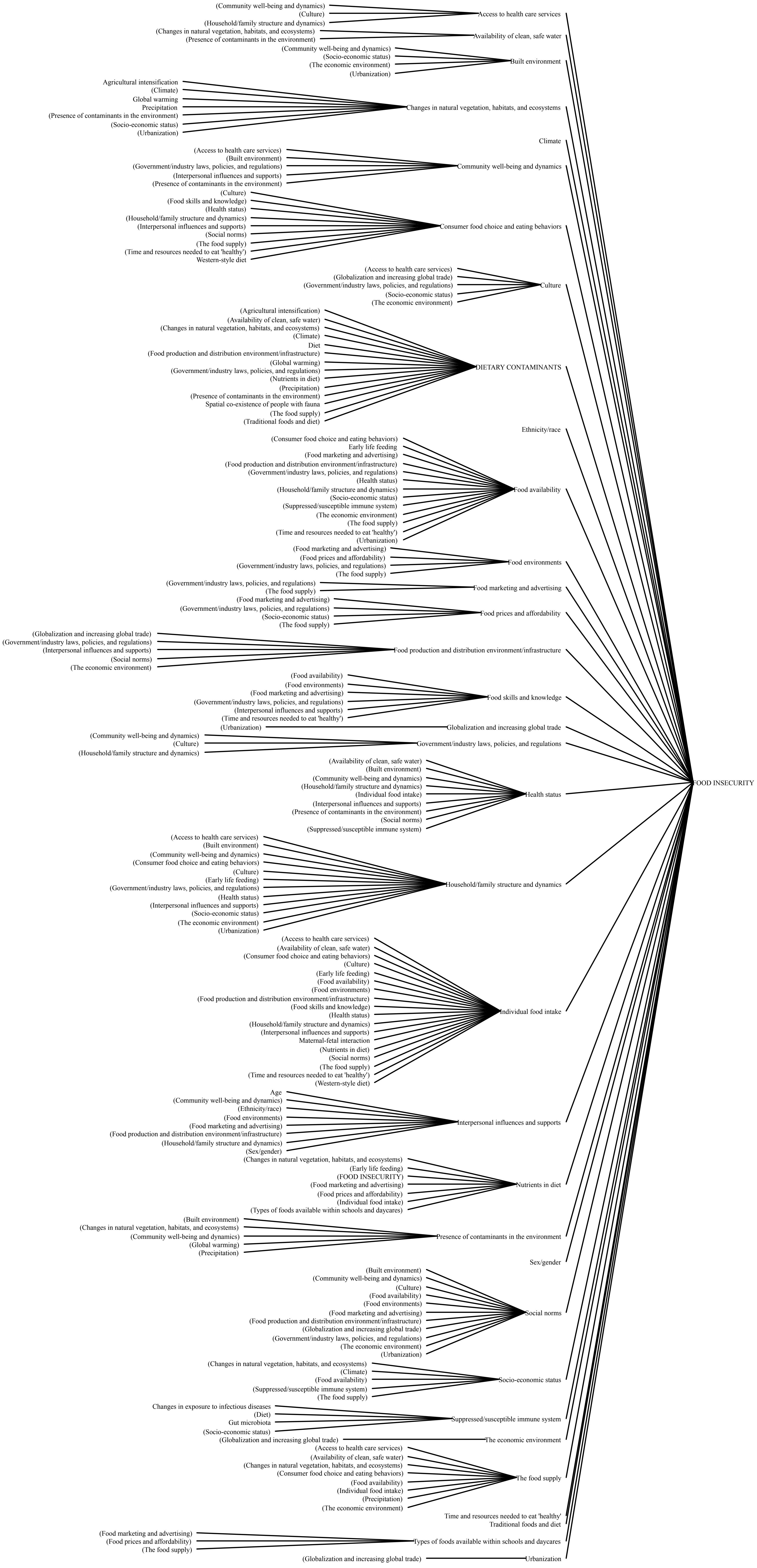
(Community well-being and dynamics)
(Culture)
(Household/family structure and dynamics)
Access to health care services
(Changes in natural vegetation, habitats, and ecosystems)
(Presence of contaminants in the environment)
Availability of clean, safe water
(Community well-being and dynamics)
(Socio-economic status)
(The economic environment)
(Urbanization)
Built environment
Agricultural intensification
(Climate)
Global warming
Precipitation
(Presence of contaminants in the environment)
(Socio-economic status)
(Urbanization)
Changes in natural vegetation, habitats, and ecosystems
Climate
(Access to health care services)
(Built environment)
(Government/industry laws, policies, and regulations)
(Interpersonal influences and supports)
(Presence of contaminants in the environment)
Community well-being and dynamics
(Culture)
(Food skills and knowledge)
(Health status)
(Household/family structure and dynamics)
(Interpersonal influences and supports)
(Social norms)
(The food supply)
(Time and resources needed to eat 'healthy')
Western-style diet
Consumer food choice and eating behaviors
(Access to health care services)
(Globalization and increasing global trade)
(Government/industry laws, policies, and regulations)
(Socio-economic status)
(The economic environment)
Culture
(Agricultural intensification)
(Availability of clean, safe water)
(Changes in natural vegetation, habitats, and ecosystems)
(Climate)
Diet
(Food production and distribution environment/infrastructure)
(Global warming)
(Government/industry laws, policies, and regulations)
(Nutrients in diet)
(Precipitation)
(Presence of contaminants in the environment)
Spatial co-existence of people with fauna
(The food supply)
(Traditional foods and diet)
DIETARY CONTAMINANTS
Ethnicity/race
(Consumer food choice and eating behaviors)
Early life feeding
(Food marketing and advertising)
(Food production and distribution environment/infrastructure)
(Government/industry laws, policies, and regulations)
(Health status)
(Household/family structure and dynamics)
(Socio-economic status)
(Suppressed/susceptible immune system)
(The economic environment)
(The food supply)
(Time and resources needed to eat 'healthy')
(Urbanization)
Food availability
(Food marketing and advertising)
(Food prices and affordability)
(Government/industry laws, policies, and regulations)
(The food supply)
Food environments
(Government/industry laws, policies, and regulations)
(The food supply)
Food marketing and advertising
(Food marketing and advertising)
(Government/industry laws, policies, and regulations)
(Socio-economic status)
(The food supply)
Food prices and affordability
(Globalization and increasing global trade)
(Government/industry laws, policies, and regulations)
(Interpersonal influences and supports)
(Social norms)
(The economic environment)
Food production and distribution environment/infrastructure
(Food availability)
(Food environments)
(Food marketing and advertising)
(Government/industry laws, policies, and regulations)
(Interpersonal influences and supports)
(Time and resources needed to eat 'healthy')
Food skills and knowledge
(Urbanization)
Globalization and increasing global trade
(Community well-being and dynamics)
(Culture)
(Household/family structure and dynamics)
Government/industry laws, policies, and regulations
(Availability of clean, safe water)
(Built environment)
(Community well-being and dynamics)
(Household/family structure and dynamics)
(Individual food intake)
(Interpersonal influences and supports)
(Presence of contaminants in the environment)
(Social norms)
(Suppressed/susceptible immune system)
Health status
FOOD INSECURITY
(Access to health care services)
(Built environment)
(Community well-being and dynamics)
(Consumer food choice and eating behaviors)
(Culture)
(Early life feeding)
(Government/industry laws, policies, and regulations)
(Health status)
(Interpersonal influences and supports)
(Socio-economic status)
(The economic environment)
(Urbanization)
Household/family structure and dynamics
(Access to health care services)
(Availability of clean, safe water)
(Consumer food choice and eating behaviors)
(Culture)
(Early life feeding)
(Food availability)
(Food environments)
(Food production and distribution environment/infrastructure)
(Food skills and knowledge)
(Health status)
(Household/family structure and dynamics)
(Interpersonal influences and supports)
Maternal-fetal interaction
(Nutrients in diet)
(Social norms)
(The food supply)
(Time and resources needed to eat 'healthy')
(Western-style diet)
Individual food intake
Age
(Community well-being and dynamics)
(Ethnicity/race)
(Food environments)
(Food marketing and advertising)
(Food production and distribution environment/infrastructure)
(Household/family structure and dynamics)
(Sex/gender)
Interpersonal influences and supports
(Changes in natural vegetation, habitats, and ecosystems)
(Early life feeding)
(FOOD INSECURITY)
(Food marketing and advertising)
(Food prices and affordability)
(Individual food intake)
(Types of foods available within schools and daycares)
Nutrients in diet
(Built environment)
(Changes in natural vegetation, habitats, and ecosystems)
(Community well-being and dynamics)
(Global warming)
(Precipitation)
Presence of contaminants in the environment
Sex/gender
(Built environment)
(Community well-being and dynamics)
(Culture)
(Food availability)
(Food environments)
(Food marketing and advertising)
(Food production and distribution environment/infrastructure)
(Globalization and increasing global trade)
(Government/industry laws, policies, and regulations)
(The economic environment)
(Urbanization)
Social norms
(Changes in natural vegetation, habitats, and ecosystems)
(Climate)
(Food availability)
(Suppressed/susceptible immune system)
(The food supply)
Socio-economic status
Changes in exposure to infectious diseases
(Diet)
Gut microbiota
(Socio-economic status)
Suppressed/susceptible immune system
(Globalization and increasing global trade)
The economic environment
(Access to health care services)
(Availability of clean, safe water)
(Changes in natural vegetation, habitats, and ecosystems)
(Consumer food choice and eating behaviors)
(Food availability)
(Individual food intake)
(Precipitation)
(The economic environment)
The food supply
Time and resources needed to eat 'healthy'
Traditional foods and diet
(Food marketing and advertising)
(Food prices and affordability)
(The food supply)
Types of foods available within schools and daycares
(Globalization and increasing global trade)
Urbanization

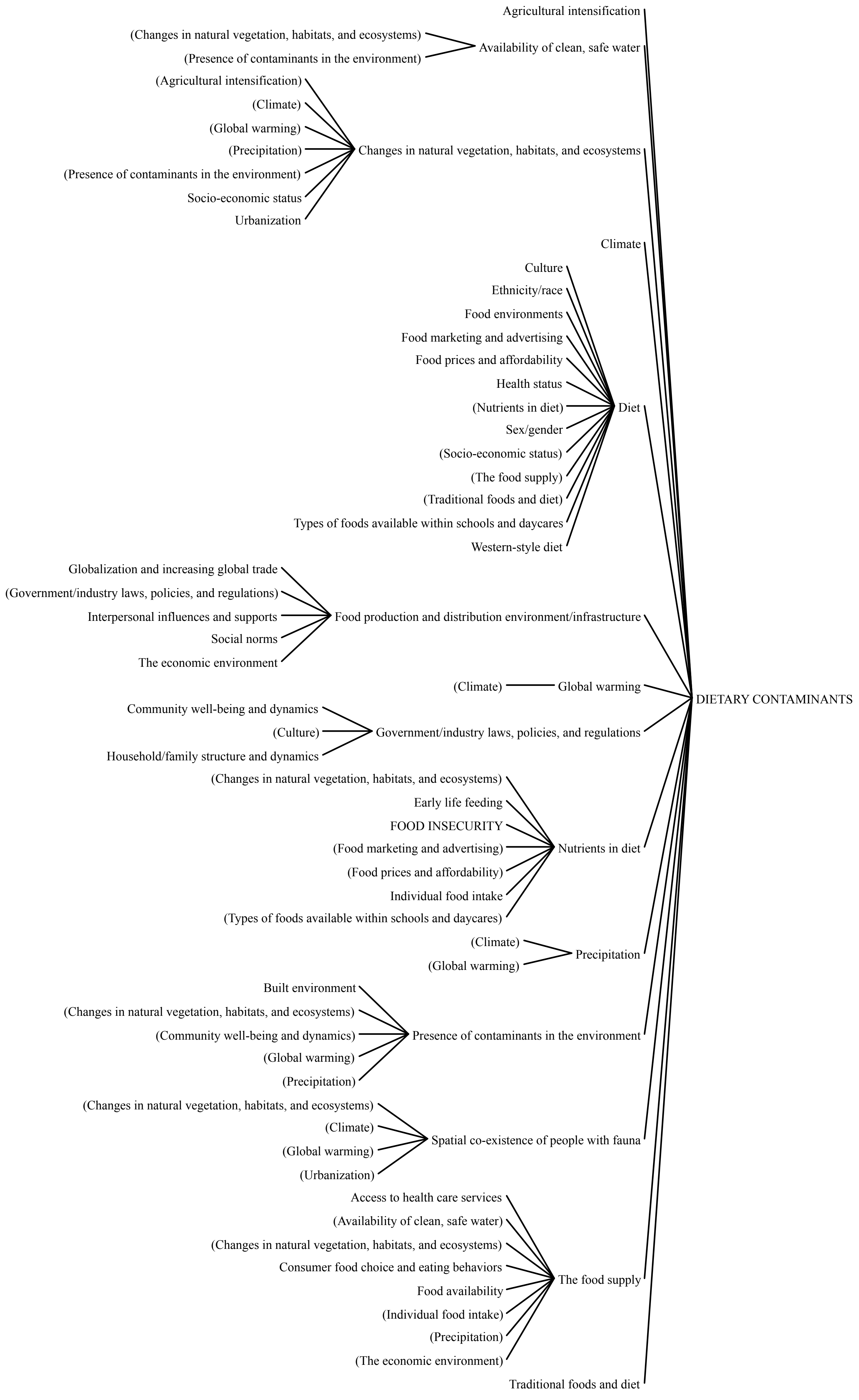
Agricultural intensification
(Changes in natural vegetation, habitats, and ecosystems)
(Presence of contaminants in the environment)
Availability of clean, safe water
(Agricultural intensification)
(Climate)
(Global warming)
(Precipitation)
(Presence of contaminants in the environment)
Socio-economic status
Urbanization
Changes in natural vegetation, habitats, and ecosystems
Climate
Culture
Ethnicity/race
Food environments
Food marketing and advertising
Food prices and affordability
Health status
(Nutrients in diet)
Sex/gender
(Socio-economic status)
(The food supply)
(Traditional foods and diet)
Types of foods available within schools and daycares
Western-style diet
Diet
Globalization and increasing global trade
(Government/industry laws, policies, and regulations)
Interpersonal influences and supports
Social norms
The economic environment
Food production and distribution environment/infrastructure
(Climate)
Global warming
DIETARY CONTAMINANTS
Community well-being and dynamics
(Culture)
Household/family structure and dynamics
Government/industry laws, policies, and regulations
(Changes in natural vegetation, habitats, and ecosystems)
Early life feeding
FOOD INSECURITY
(Food marketing and advertising)
(Food prices and affordability)
Individual food intake
(Types of foods available within schools and daycares)
Nutrients in diet
(Climate)
(Global warming)
Precipitation
Built environment
(Changes in natural vegetation, habitats, and ecosystems)
(Community well-being and dynamics)
(Global warming)
(Precipitation)
Presence of contaminants in the environment
(Changes in natural vegetation, habitats, and ecosystems)
(Climate)
(Global warming)
(Urbanization)
Spatial co-existence of people with fauna
Access to health care services
(Availability of clean, safe water)
(Changes in natural vegetation, habitats, and ecosystems)
Consumer food choice and eating behaviors
Food availability
(Individual food intake)
(Precipitation)
(The economic environment)
The food supply
Traditional foods and diet
